# Updated checklist of the birds of Calabria (southern Italy)

**DOI:** 10.3897/BDJ.14.e195967

**Published:** 2026-07-13

**Authors:** Salvatore Urso, Giuseppe Martino, Eugenio Muscianese, Manuela Policastrese, Mario Pucci, Maurizio Vena, Pierpaolo Storino

**Affiliations:** 1 Stazione Ornitologica Calabrese (StOrCal), Cosenza, Italy Stazione Ornitologica Calabrese (StOrCal) Cosenza Italy

**Keywords:** AERC, avifauna, birds, Calabria, checklist, Italy, southern Italy

## Abstract

**Background:**

Information on the avifauna of Calabria is still incomplete, with much of it contained in isolated studies, local reports and unpublished observations. More than thirty years have passed since the publication of the first regional checklist. The last ten years have seen an increase in fieldwork and collaboration amongst local ornithologists, now coordinated by the Stazione Ornitologica Calabrese (StOrCal).

**New information:**

We provide an updated checklist of the birds of Calabria, revised through 31 December 2025, representing an increase of 43 species compared to the previous regional checklist of 320 species. The total number of taxa is 363 species and nine subspecies, divided as follows: 336 AERC category A, 12 B, 7 C, 5 AC and 3 E. General status codes were assigned to 258 regular species, 20 irregular species, 64 accidental/vagrant species and 21 historical species. Breeding status was assigned to 143 regularly breeding species, eight irregular, three occasional and four former breeding species. A total of 125 species are included in Annex I of Directive 2009/147/EC. National IUCN categories are known for 230 species, including nine Critically Endangered, 21 Endangered and 32 Vulnerable species. Taxonomy and status coding are from the national CISO–COI checklist.

## Introduction

Information on the avifauna of Calabria is still incomplete, with much of it contained in isolated studies, local reports and unpublished observations. More than thirty years have passed since the publication of the first regional checklist (Scebba et al. 1993). The last ten years have seen an increase in fieldwork and collaboration amongst local ornithologists, now coordinated by the Stazione Ornitologica Calabrese (StOrCal). Here, we update the historical checklist by critically incorporating newer sources and records and by using a standardised code for occurrence and breeding status that is fully compatible with the national CISO–COI checklist (Baccetti et al. 2021).

## Materials and methods

### Study area

Calabria is the southernmost mainland region of the Italian peninsula, bounded by the Tyrrhenian Sea on the west and the Ionian Sea on the east. The region covers approximately 15,222 km² and extends about 248 km from north to south, with a maximum width of about 110 km. It is administratively divided into five provinces (Cosenza, Catanzaro, Crotone, Reggio Calabria and Vibo Valentia). The regional coastline extends for approximately 789 km, representing about one-tenth of the entire Italian marine boundary.

Calabria spans a marked elevational gradient, from sea level to 2,267 m a.s.l. at Serra Dolcedorme in the Pollino Massif, the highest peak of the region. The territory is predominantly mountainous and hilly, with plains covering less than 10% of the surface. Three main mountain systems characterise the regional landscape: the Pollino Massif in the north, the Sila Plateau in the centre (Botte Donato, 1,928 m a.s.l.) and the Aspromonte Massif in the south (Montalto, 1,955 m a.s.l.). These uplands host extensive forests (beech, silver fir, Calabrian black pine, Bosnian pine) and montane grasslands, providing habitat for breeding raptors, woodpeckers and passerines of Mediterranean–Apennine affinity.

The coastal and lowland sectors include agricultural plains (Sibari, Lamezia, Gioia Tauro, Crotone), coastal wetlands, river mouths and sandy beaches, which are important for wintering and migrating waterbirds and shorebirds. The major river systems (Crati, Neto, Lao, Mesima) and the associated transitional environments support diverse waterbird communities. The Mediterranean climate, the central position within the central Mediterranean migratory flyway and the proximity to Sicily and the African continent make Calabria a strategically important region for bird migration and a regular receiver of vagrant species.

**Figure 1.**
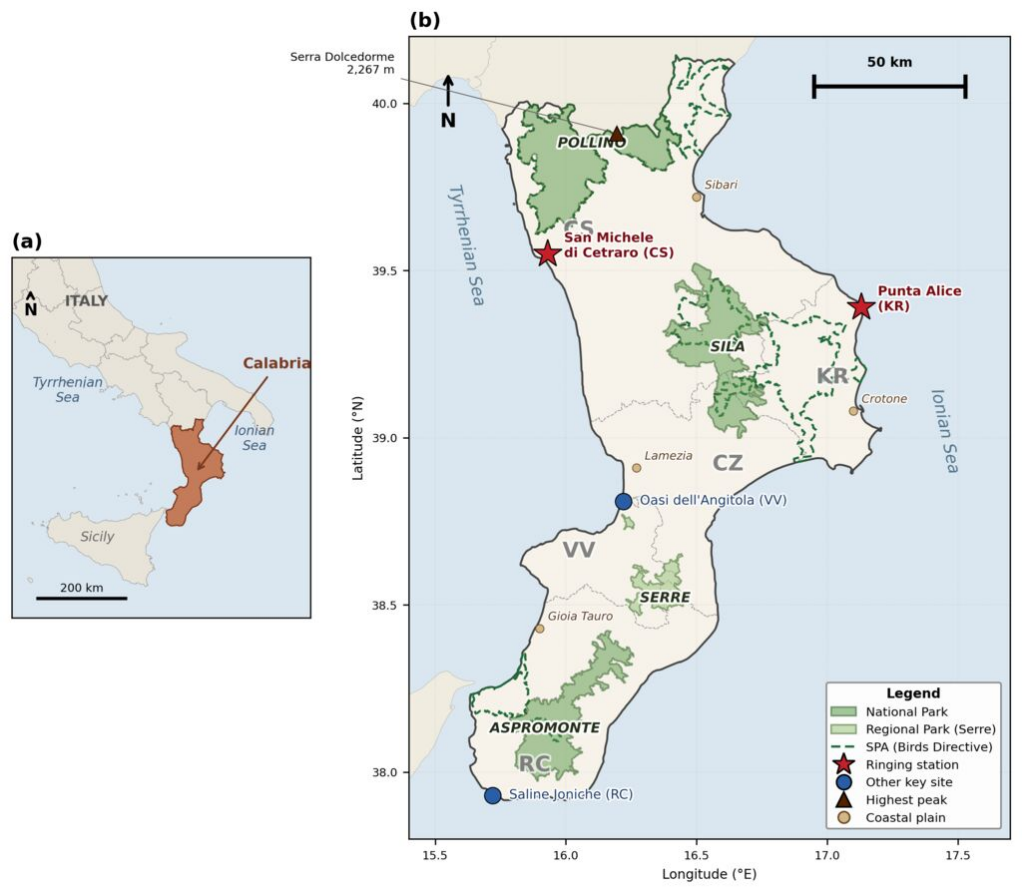
Geographic setting of Calabria (southern Italy). **(a)** Location of the region within the central Mediterranean, between the Tyrrhenian and Ionian seas; **(b)** Main mountain massifs (Pollino, Sila, Serre, Aspromonte), coastal plains, protected areas and the key bird observation and ringing sites mentioned in the present checklist. National parks, the regional park and Special Protection Areas (SPAs, designated under Directive 2009/147/EC) are shown together with the highest peak of the region (Serra Dolcedorme, 2,267 m a.s.l.). Protected-area boundaries are sourced from the World Database on Protected Areas (WDPA; UNEP-WCMC & IUCN, June 2026); administrative boundaries are derived from ISTAT.

Calabria hosts an extensive network of protected areas. These include three national parks (Pollino, Sila and Aspromonte), one regional park (Serre), several state and regional nature reserves and 185 Natura 2000 sites covering approximately 19% of the regional territory, of which six are Special Protection Areas (SPAs), designated under Directive 2009/147/EC. The location of the main mountain massifs, protected areas and key bird observation and ringing sites mentioned in the present checklist is shown in Fig. 1.

### Data sources and inclusion criteria

The starting point for this updated checklist was the regional checklist by Scebba et al. (1993), which we critically revised and updated with published and unpublished sources available to us up to 31 December 2025. We carried out a systematic review of the regional ornithological literature, including peer-reviewed papers, technical reports, theses, ringing scheme bulletins, conference proceedings and local naturalistic journals, which represent the main bibliographic sources for the avifauna of Calabria. Full references are listed in the References section.

In addition to the published literature, we consulted the citizen-science platforms ORNITHO.it, eBird and iNaturalist, which represent the main repositories of observational data for Italian avifauna. The majority of regional records uploaded to these platforms originated from the authors of the present checklist. When records from other contributors were considered relevant for inclusion, the original observers were contacted directly by the authors and the corresponding data were used only after explicit authorisation by the observer. Records reported on these platforms were considered only when supported by adequate documentation (photograph, sound recording or detailed field notes) and, in the case of rare or unusual records, only when validated by the relevant national review body, namely the Italian Rarities Committee (Commissione Ornitologica Italiana, COI).

A substantial portion of the data presented here derives from the long-term fieldwork conducted throughout Calabria, including standardised bird ringing at coastal and inland stations (notably Punta Alice, KR and San Michele di Cetraro, CS), targeted breeding surveys, wintering counts and systematic monitoring of key wetlands and protected areas. Unpublished records provided by members of the Stazione Ornitologica Calabrese (StOrCal) and by the wider regional birdwatching community were accepted following an internal review by the authors, which assessed the available evidence on a case-by-case basis.

For each taxon, inclusion in the checklist required at least one of the following types of supporting evidence: (i) a published reference in peer-reviewed or grey literature; (ii) a museum specimen with verifiable collection data; (iii) a photograph, video or audio recording archived in a public repository or held by the authors; (iv) a ringed individual with full ringing data; (v) detailed field notes from experienced observers, especially when corroborated by independent records.

Records considered insufficiently documented or whose attribution to Calabria could not be verified were excluded. For accidental and vagrant taxa, all available record-level details (year, number of individuals, province, observer or literature sources) are reported in the Notes field of the Checklists section. Province codes follow standard Italian administrative abbreviations for Calabria: CS = Cosenza, CZ = Catanzaro, KR = Crotone, RC = Reggio Calabria and VV = Vibo Valentia.

A small number of records included in the present checklist derive from Scebba et al. (1993) and are reported there without precise locality or date information. These records were originally based on museum specimens, contemporary technical documentation or first-hand observations by ornithologists active in the region at the time of the compilation. We retained these records following the practice adopted by Scebba et al. (1993) and consistent with the AERC category A criterion. For full transparency, such records are explicitly flagged in the Notes field of the Checklists section as having undocumented date and/or locality information, allowing future researchers to re-evaluate their status should new evidence emerge.

### Taxonomy and status coding

Taxonomy, systematic order and the coding of AERC category, general status and breeding status follow the CISO–COI checklist of Italian birds (Baccetti et al. 2021). The combined code comprises the AERC category letter (A, B, C, D, E or AC) and two digits indicating general status and breeding status. Category and code definitions are shown in Table 1. We also indicate whether taxa are listed in Annex I of Directive 2009/147/EC (Birds Directive) and report the national IUCN category from the Red List of breeding birds in Italy (Gustin et al. 2021) where available. English common names are aligned with the Global Avian Checklist (Christidis et al. 2025).

Taxonomic concepts and the systematic order of orders, families and species follow the CISO–COI checklist of Italian birds (Baccetti et al. 2021). English common names, however, are aligned with the Global Avian Checklist (Christidis et al. 2025), which constitutes the current international standard for English vernacular nomenclature. We acknowledge that the two sources are not always fully concordant, particularly regarding recent genus-level revisions (e.g. the splitting of *Sylvia* into *Sylvia* and *Curruca*; the placement of certain species in *Ixobrychus*/*Botaurus*, *Bubulcus*/*Ardea*, *Charadrius*/*Anarhynchus*/*Thinornis* and within the Laridae). In all such cases, scientific names follow the CISO–COI checklist (Baccetti et al. 2021), which represents the reference framework for Italian regional and national avifaunistic synthesis. Where AviList adopts a different generic placement, the species' identity is unambiguously preserved through the binomial name, so that the two systems remain fully interoperable at the species level.

## Data resources

The checklist dataset supporting this publication is openly available on Zenodo (Urso et al. 2026) https://doi.org/10.5281/zenodo.19184234.

## Checklists

### Updated checklist of the birds of Calabria (southern Italy)

#### Coturnix coturnix

2FDFE9C5-FF81-5FF2-AB9C-2E4477222414

##### Conservation status

DD

##### Distribution

Calabria, southern Italy

##### Notes

Code: A11.

#### Alectoris graeca

A4F26291-7BEC-5A06-8E4C-692248E83CF6

##### Conservation status

VU

##### Distribution

Calabria, southern Italy

##### Notes

Code: AC11; Annex I Birds Directive (2009/147/EC).

#### Alectoris chukar

3A98051A-68E5-5BDC-9DC1-23C57AA9B229

##### Distribution

Calabria, southern Italy

##### Notes

Code: C30.

#### Alectoris rufa

76F795F5-1A7B-5545-B74C-873AA6F050EF

##### Conservation status

DD

##### Distribution

Calabria, southern Italy

##### Notes

Code: C30.

#### Phasianus colchicus

3C40B0F0-7925-5C46-83B2-417FAA366C0E

##### Conservation status

NA

##### Distribution

Calabria, southern Italy

##### Notes

Code: C11.

#### Perdix perdix

F56CCFBC-2672-5252-B43E-D696648A17E3

##### Conservation status

NT

##### Distribution

Calabria, southern Italy

##### Notes

Code: AC24.

#### Oxyura jamaicensis

6EAA6CB6-F912-5775-A04C-1D33E96CEC60

##### Distribution

Calabria, southern Italy

##### Notes

Code: C30.

#### Oxyura leucocephala

69381732-F5E9-549F-8787-996CCE04CDBF

##### Conservation status

RE

##### Distribution

Calabria, southern Italy

##### Notes

Code: A30; Annex I Birds Directive (2009/147/EC); Accidental record(s): one record (CS 1986), in Scebba et al. (1993).

#### Cygnus olor

AF7CF6A9-0CA6-5494-9133-96F8AE3E6209

##### Conservation status

LC

##### Distribution

Calabria, southern Italy

##### Notes

Code: AC20.

#### Cygnus cygnus

EE29D23B-9E69-58A3-B8BA-A363A87395BC

##### Distribution

Calabria, southern Italy

##### Notes

Code: A40; Annex I Birds Directive (2009/147/EC).

#### Branta leucopsis

DF534394-299C-58EA-8BF8-B1EC5D3D6474

##### Distribution

Calabria, southern Italy

##### Notes

Code: E30; Annex I Birds Directive (2009/147/EC).

#### Anser anser

C577E374-AFA7-54D4-B024-0249517F685A

##### Conservation status

LC

##### Distribution

Calabria, southern Italy

##### Notes

Code: AC20.

#### Anser fabalis

4723ECF4-775D-5829-A4A3-9B7846715C9B

##### Distribution

Calabria, southern Italy

##### Notes

Code: A30; Accidental record(s): one record (date and locality unknown), in Scebba et al. (1993).

#### Anser albifrons

96961DED-15A1-55C9-8A68-6C27D66C33D0

##### Distribution

Calabria, southern Italy

##### Notes

Code: A30; Accidental record(s): three records (CS 1984, 1986; VV 2021), historical records in Scebba et al. (1993); most recent record by Giuseppe Paolillo from https://www.ilvibonese.it/ambiente/loca-lombardella-torna-in-calabria-dopo-40-anni-avvistata-nelloasi-dellangitola-uk8187lj.

#### Clangula hyemalis

8CAB6CFE-43DE-5B07-A36D-EAA706F9C1BE

##### Distribution

Calabria, southern Italy

##### Notes

Code: A30; Accidental record(s): one record (KR 2002), in Zenatello et al. (2014).

#### Melanitta fusca

B404A5F4-AA44-556B-832A-933875C29FCB

##### Distribution

Calabria, southern Italy

##### Notes

Code: A40.

#### Melanitta nigra

F8DB1B6B-0DDB-5483-A0C1-489D968E2510

##### Distribution

Calabria, southern Italy

##### Notes

Code: A30; Accidental record(s): one record (CS 1979), in Scebba et al. (1993).

#### Bucephala clangula

513BF571-8B4E-501C-B353-A8EC08BE8E26

##### Distribution

Calabria, southern Italy

##### Notes

Code: A30; Accidental record(s): one record (RC, 5 January 2011), by Federico Capitani.

#### Mergellus albellus

5B52C9E0-617D-551C-84B5-F1F83F09F823

##### Distribution

Calabria, southern Italy

##### Notes

Code: A40; Annex I Birds Directive (2009/147/EC).

#### Mergus merganser

91601538-1B88-5135-B7A8-E34A74EABD95

##### Conservation status

LC

##### Distribution

Calabria, southern Italy

##### Notes

Code: A30; Accidental record(s): two records (KR 1971; CS 1985), in Scebba et al. (1993).

#### Mergus serrator

F57B8E55-9FE4-5CCB-B0E6-D835E9AC5EF3

##### Distribution

Calabria, southern Italy

##### Notes

Code: A20.

#### Alopochen aegyptiaca

DA399A26-BA70-5C88-8127-8F0DDFB19F2C

##### Distribution

Calabria, southern Italy

##### Notes

Code: E30.

#### Tadorna tadorna

49BF903F-CC8B-5CF4-AE3E-ABA14BAE6241

##### Conservation status

VU

##### Distribution

Calabria, southern Italy

##### Notes

Code: A13.

#### Tadorna ferruginea

FAF33C50-F1E9-52AC-9C08-49117A5CA61F

##### Distribution

Calabria, southern Italy

##### Notes

Code: A30; Annex I Birds Directive (2009/147/EC); Accidental record(s): six records (CS 1993 [2]; RC 1994; KR 2004; RC 2013; CS 2018), historical records in Scebba et al. (1993); records after 1994 reported by the authors of the present checklist.

#### Aix galericulata

86390D74-4FA4-5127-8104-EAC0673FBFF7

##### Distribution

Calabria, southern Italy

##### Notes

Code: E30.

#### Marmaronetta angustirostris

433E2496-807D-5C2F-834E-8F2230257330

##### Conservation status

EN

##### Distribution

Calabria, southern Italy

##### Notes

Code: A30; Annex I Birds Directive (2009/147/EC); Accidental record(s): one record (KR, 22 September 2017), by Mario Pucci.

#### Netta rufina

7AE34226-6EAD-5020-89D4-CC41BDDFB08E

##### Conservation status

VU

##### Distribution

Calabria, southern Italy

##### Notes

Code: A20.

#### Aythya ferina

3C7CAD5C-9C46-5038-A69E-808D4D0FCFE7

##### Conservation status

VU

##### Distribution

Calabria, southern Italy

##### Notes

Code: A10.

#### Aythya nyroca

42E1A23B-A37D-501E-AB67-5838F0801F5A

##### Conservation status

EN

##### Distribution

Calabria, southern Italy

##### Notes

Code: A10; Annex I Birds Directive (2009/147/EC).

#### Aythya fuligula

5BF490CB-9D93-5B77-988E-0C334185D258

##### Conservation status

VU

##### Distribution

Calabria, southern Italy

##### Notes

Code: A10.

#### Aythya marila

AEF8EBDB-F727-521B-9D07-5D9DB477B7CE

##### Distribution

Calabria, southern Italy

##### Notes

Code: A40.

#### Spatula querquedula

81ABB21B-7345-5EA9-9BEE-58D3DB4C6E92

##### Conservation status

VU

##### Distribution

Calabria, southern Italy

##### Notes

Code: A10.

#### Spatula clypeata

21B7EA64-726F-5524-BFD5-CEE60FA7F264

##### Conservation status

VU

##### Distribution

Calabria, southern Italy

##### Notes

Code: A10.

#### Mareca strepera

58964050-992B-53E8-A236-F3339B3BD954

##### Conservation status

NT

##### Distribution

Calabria, southern Italy

##### Notes

Code: A10.

#### Mareca penelope

63E62C08-F514-5EAD-B899-957191BBA863

##### Conservation status

NA

##### Distribution

Calabria, southern Italy

##### Notes

Code: A10.

#### Anas platyrhynchos

20886A0E-A320-5A4D-99F5-0DAFED02F0CD

##### Conservation status

LC

##### Distribution

Calabria, southern Italy

##### Notes

Code: A11.

#### Anas acuta

3FB93DB0-C499-54C3-9A40-A90F13553306

##### Conservation status

NA

##### Distribution

Calabria, southern Italy

##### Notes

Code: A10.

#### Anas crecca

54C350A4-466B-5605-A546-7B1D1B428FA5

##### Conservation status

EN

##### Distribution

Calabria, southern Italy

##### Notes

Code: A10.

#### Tachybaptus ruficollis

8AA39C18-1A33-54D6-AAB9-155D71F8AECC

##### Conservation status

LC

##### Distribution

Calabria, southern Italy

##### Notes

Code: A11.

#### Podiceps grisegena

02A591C1-DFB4-57D9-8984-B341232E0A5A

##### Distribution

Calabria, southern Italy

##### Notes

Code: A40.

#### Podiceps cristatus

BAA8F920-58FB-5C8C-9C88-639AE29EC68E

##### Conservation status

LC

##### Distribution

Calabria, southern Italy

##### Notes

Code: A11.

#### Podiceps auritus

87F7B017-FB81-589D-A91E-8F4560449E4A

##### Distribution

Calabria, southern Italy

##### Notes

Code: A30; Annex I Birds Directive (2009/147/EC); Accidental record(s): one record (KR, 1 January 2022), by Mario Pucci.

#### Podiceps nigricollis

0C92F640-BFDE-5E33-BFAB-55705EDD349F

##### Conservation status

NA

##### Distribution

Calabria, southern Italy

##### Notes

Code: A12.

#### Phoenicopterus roseus

F09AC3E5-FFA5-5A70-B16B-D8265E522EB1

##### Conservation status

LC

##### Distribution

Calabria, southern Italy

##### Notes

Code: A10; Annex I Birds Directive (2009/147/EC).

#### Columba livia

69ECE59E-F3AC-5CB9-B8C5-827AC6B5ADFF

##### Conservation status

DD

##### Distribution

Calabria, southern Italy

##### Notes

Code: A11.

#### Columba oenas

B0344041-F95E-5C0C-A1E7-FEC5E0DAD560

##### Conservation status

DD

##### Distribution

Calabria, southern Italy

##### Notes

Code: A14.

#### Columba palumbus

03662E81-B1CC-5E45-9498-DDCC5565F33A

##### Conservation status

LC

##### Distribution

Calabria, southern Italy

##### Notes

Code: A11.

#### Streptopelia turtur

7EFAA38A-650E-58C2-9B56-072E66FE102D

##### Conservation status

LC

##### Distribution

Calabria, southern Italy

##### Notes

Code: A11.

#### Streptopelia decaocto

AA1EF8A8-51EF-5E8E-8378-828458EFA7B7

##### Conservation status

LC

##### Distribution

Calabria, southern Italy

##### Notes

Code: A11.

#### Spilopelia senegalensis

C1FEA3C6-886B-5AF1-BBCB-FDC24F6B45BF

##### Conservation status

NT

##### Distribution

Calabria, southern Italy

##### Notes

Code: A30; Accidental record(s): one record (RC, 25 October 2016), by Giuseppe Martino.

#### Caprimulgus europaeus

180BE9CF-043D-5D0C-99E3-CC6441E255AD

##### Conservation status

LC

##### Distribution

Calabria, southern Italy

##### Notes

Code: A11; Annex I Birds Directive (2009/147/EC).

#### Tachymarptis melba

D3DA33E9-FB6B-5145-A4E8-473B7B9D2817

##### Conservation status

LC

##### Distribution

Calabria, southern Italy

##### Notes

Code: A11.

#### Apus caffer

4DDD7FFE-6B0F-518F-A9B3-B9119701F4C2

##### Distribution

Calabria, southern Italy

##### Notes

Code: A22; Annex I Birds Directive (2009/147/EC).

#### Apus pallidus

8D99AC09-D23A-5730-8526-125C3131F4AF

##### Conservation status

LC

##### Distribution

Calabria, southern Italy

##### Notes

Code: A11.

#### Apus apus

F67203CD-E3CD-5EFD-BA7D-6F826689BB48

##### Conservation status

LC

##### Distribution

Calabria, southern Italy

##### Notes

Code: A11.

#### Clamator glandarius

B31ADE17-5B99-5D89-931F-3D4BE9FC0FC3

##### Conservation status

LC

##### Distribution

Calabria, southern Italy

##### Notes

Code: A10.

#### Cuculus canorus

1AD77AE7-928F-56CF-9078-2D0A5CA9F9F4

##### Conservation status

NT

##### Distribution

Calabria, southern Italy

##### Notes

Code: A11.

#### Rallus aquaticus

CFFF6D79-74EC-5C86-83EB-E2F8E3F5C67D

##### Conservation status

LC

##### Distribution

Calabria, southern Italy

##### Notes

Code: A11.

#### Crex crex

25BF74CC-F1E5-5F41-811D-B2A8F7A5393E

##### Conservation status

VU

##### Distribution

Calabria, southern Italy

##### Notes

Code: A10; Annex I Birds Directive (2009/147/EC).

#### Porzana porzana

D8BB5AA8-7022-5943-897A-F110CFA0DDA6

##### Conservation status

CR

##### Distribution

Calabria, southern Italy

##### Notes

Code: A10; Annex I Birds Directive (2009/147/EC).

#### Zapornia parva

5A311D8D-2DC7-53B0-8CD6-125161856DE6

##### Conservation status

CR

##### Distribution

Calabria, southern Italy

##### Notes

Code: A10; Annex I Birds Directive (2009/147/EC).

#### Zapornia pusilla

58B5C7A8-E994-5B15-8C81-F2E719A45FBE

##### Conservation status

NA

##### Distribution

Calabria, southern Italy

##### Notes

Code: A30; Annex I Birds Directive (2009/147/EC); Accidental record(s): two records; the most recent (CS, 5 March 2017) by Cataldo Lattieri (via Domenico Verducci).

#### Porphyrio porphyrio

F7A5C3A4-7C8E-56E8-A358-4766695239C2

##### Conservation status

NT

##### Distribution

Calabria, southern Italy

##### Notes

Code: A33; Annex I Birds Directive (2009/147/EC).

#### Gallinula chloropus

468F2E00-EEA3-53D5-861C-B680F2E8D8A9

##### Conservation status

LC

##### Distribution

Calabria, southern Italy

##### Notes

Code: A11.

#### Fulica atra

B11919EF-4F47-5975-8122-C6EDCA52E676

##### Conservation status

LC

##### Distribution

Calabria, southern Italy

##### Notes

Code: A11.

#### Grus grus

96E2FBFA-4080-5198-BB70-EA21F7EADB17

##### Conservation status

RE

##### Distribution

Calabria, southern Italy

##### Notes

Code: A10; Annex I Birds Directive (2009/147/EC).

#### Tetrax tetrax

1B1CD567-73E1-5709-96E9-B68F35ECCC5B

##### Conservation status

EN

##### Distribution

Calabria, southern Italy

##### Notes

Code: B40; Annex I Birds Directive (2009/147/EC).

#### Otis tarda

FCB37950-2F84-5F4C-BB4A-529DB646C891

##### Distribution

Calabria, southern Italy

##### Notes

Code: B40; Annex I Birds Directive (2009/147/EC).

#### Gavia stellata

039A41CE-2964-5D27-93EB-C8FF5AA5223F

##### Distribution

Calabria, southern Italy

##### Notes

Code: A20; Annex I Birds Directive (2009/147/EC).

#### Gavia arctica

6D321210-4DE7-5289-881D-9406723D4D9F

##### Distribution

Calabria, southern Italy

##### Notes

Code: A10; Annex I Birds Directive (2009/147/EC).

#### Hydrobates pelagicus

FD2C581A-C591-559D-8B63-2629EDC11EE3

##### Conservation status

NT

##### Distribution

Calabria, southern Italy

##### Notes

Code: A10; Annex I Birds Directive (2009/147/EC).

#### Calonectris diomedea

72DACD3D-1CE1-5468-9385-55F29A6EA206

##### Conservation status

LC

##### Distribution

Calabria, southern Italy

##### Notes

Code: A10; Annex I Birds Directive (2009/147/EC).

#### Puffinus yelkouan

1583765D-7216-5997-982B-8DDD5CF1BA8C

##### Conservation status

DD

##### Distribution

Calabria, southern Italy

##### Notes

Code: A10; Annex I Birds Directive (2009/147/EC).

#### Ciconia nigra

8612E399-B637-5A1F-B8DB-0EC32027AC0A

##### Conservation status

EN

##### Distribution

Calabria, southern Italy

##### Notes

Code: A11; Annex I Birds Directive (2009/147/EC).

#### Ciconia ciconia

ED08417C-E3FC-59AE-96EC-4C00302AC455

##### Conservation status

LC

##### Distribution

Calabria, southern Italy

##### Notes

Code: A11; Annex I Birds Directive (2009/147/EC).

#### Platalea leucorodia

DFDE5C90-3756-5EB9-948C-DC33D2F170D4

##### Conservation status

NT

##### Distribution

Calabria, southern Italy

##### Notes

Code: A10; Annex I Birds Directive (2009/147/EC).

#### Threskiornis aethiopicus

0D6539D1-136E-5043-85EF-39E73B99B242

##### Distribution

Calabria, southern Italy

##### Notes

Code: C20. Italian records refer to free-ranging individuals originating from escapes from zoological collections and waterfowl parks, with subsequent local naturalisation, particularly in north-western Italy (Piedmont). Calabrian observations correspond to dispersing individuals from these naturalised populations.

#### Plegadis falcinellus

DBF5FB2A-B297-53F8-A978-C74D1319BDFB

##### Conservation status

VU

##### Distribution

Calabria, southern Italy

##### Notes

Code: A10; Annex I Birds Directive (2009/147/EC).

#### Geronticus eremita

994288BB-CA54-51F8-A1AB-F38D7026E06B

##### Distribution

Calabria, southern Italy

##### Notes

Code: C20. Italian records of this species refer to free-ranging individuals associated with re-introduction and conservation-breeding projects in central Europe and Italy, including the LIFE+ Reason for Hope programme (Austria, Germany and Italy) and the long-established colony at the Oasi dei Quadris di Fagagna (Friuli, north-eastern Italy), which maintains both free-flying and captive birds. Calabrian observations correspond to individuals dispersing or escaping from these populations.

#### Botaurus stellaris

1D94D48A-0062-5268-BBEA-8A285BD0D2C5

##### Conservation status

EN

##### Distribution

Calabria, southern Italy

##### Notes

Code: A10; Annex I Birds Directive (2009/147/EC).

#### Ixobrychus minutus

4B81B086-64F1-5685-B98C-C8899C526743

##### Conservation status

VU

##### Distribution

Calabria, southern Italy

##### Notes

Code: A11; Annex I Birds Directive (2009/147/EC).

#### Nycticorax nycticorax

D8EE3017-4D81-5B08-BDA1-2D656DE2BD54

##### Conservation status

LC

##### Distribution

Calabria, southern Italy

##### Notes

Code: A11; Annex I Birds Directive (2009/147/EC).

#### Ardeola ralloides

2B469A62-4A15-5372-A89B-D5D0016869F8

##### Conservation status

NT

##### Distribution

Calabria, southern Italy

##### Notes

Code: A12; Annex I Birds Directive (2009/147/EC).

#### Bubulcus ibis

83CDF118-F594-5FC6-BAAD-2B83912504A0

##### Conservation status

LC

##### Distribution

Calabria, southern Italy

##### Notes

Code: A12.

#### Ardea cinerea

56E704EF-ACDF-547D-942F-550981AEEFB3

##### Conservation status

LC

##### Distribution

Calabria, southern Italy

##### Notes

Code: A10.

#### Ardea purpurea

FEC6496E-0C9A-5B86-BA85-BF3D88CE8BDF

##### Conservation status

LC

##### Distribution

Calabria, southern Italy

##### Notes

Code: A10; Annex I Birds Directive (2009/147/EC).

#### Ardea alba

4FBC3B2C-728F-5C35-AD5E-2876DD3DFF9E

##### Conservation status

NT

##### Distribution

Calabria, southern Italy

##### Notes

Code: A10; Annex I Birds Directive (2009/147/EC).

#### Egretta garzetta

CAFA1C96-C1A7-5053-AA09-AD593580EEB5

##### Conservation status

LC

##### Distribution

Calabria, southern Italy

##### Notes

Code: A11; Annex I Birds Directive (2009/147/EC).

#### Egretta gularis

50CCBA23-C1BC-53B8-A1D6-12C48EE56AEF

##### Distribution

Calabria, southern Italy

##### Notes

Code: A30; Accidental record(s): one record (RC 1975), in Scebba et al. (1993).

#### Pelecanus crispus

59136139-C836-50D4-955A-C52429755A8C

##### Distribution

Calabria, southern Italy

##### Notes

Code: A40; Annex I Birds Directive (2009/147/EC).

#### Pelecanus onocrotalus

CF66BA85-F781-567F-A822-2DF25BCF9A27

##### Distribution

Calabria, southern Italy

##### Notes

Code: A40; Annex I Birds Directive (2009/147/EC).

#### Morus bassanus

30AE57E4-7248-5C13-B98C-11E4EE1ED7CC

##### Conservation status

NA

##### Distribution

Calabria, southern Italy

##### Notes

Code: A10.

#### Microcarbo pygmaeus

F5916F4E-3DE1-54FC-A35D-7CB0E9BE4572

##### Conservation status

LC

##### Distribution

Calabria, southern Italy

##### Notes

Code: A22; Annex I Birds Directive (2009/147/EC).

#### Gulosus aristotelis

478F36C3-79CF-5268-9BD0-C4E5DCE3508A

##### Conservation status

LC

##### Distribution

Calabria, southern Italy

##### Notes

Code: A30; Annex I Birds Directive (2009/147/EC); Accidental record(s): one record (date and locality unknown) in Scebba et al. (1993).

#### Phalacrocorax carbo

79032403-43E2-53F9-BC5E-7722BCD44E08

##### Conservation status

LC

##### Distribution

Calabria, southern Italy

##### Notes

Code: A12.

#### Burhinus oedicnemus

06A6F748-704F-517A-8973-0E725AEFF518

##### Conservation status

LC

##### Distribution

Calabria, southern Italy

##### Notes

Code: A11; Annex I Birds Directive (2009/147/EC).

#### Haematopus ostralegus

7BD0AB09-F8FA-55A1-9F18-C17431627852

##### Conservation status

VU

##### Distribution

Calabria, southern Italy

##### Notes

Code: A10; Annex I Birds Directive (2009/147/EC).

#### Recurvirostra avosetta

242EF985-827F-5C1F-A92E-1EB939540611

##### Conservation status

LC

##### Distribution

Calabria, southern Italy

##### Notes

Code: A10; Annex I Birds Directive (2009/147/EC).

#### Himantopus himantopus

B6E2AF34-AA2B-566B-92AF-3407A7384ECE

##### Conservation status

LC

##### Distribution

Calabria, southern Italy

##### Notes

Code: A11; Annex I Birds Directive (2009/147/EC).

#### Pluvialis squatarola

58A19F5F-68D1-5594-A974-FD3DFC0FD9C1

##### Distribution

Calabria, southern Italy

##### Notes

Code: A10.

#### Pluvialis apricaria

C95B3D50-6F41-54CB-8616-C552FEF028E2

##### Distribution

Calabria, southern Italy

##### Notes

Code: A10; Annex I Birds Directive (2009/147/EC).

#### Pluvialis fulva

73398506-32CB-5687-9BDB-F98BC3129BCE

##### Distribution

Calabria, southern Italy

##### Notes

Code: B40.

#### Eudromias morinellus

64E8F372-679F-5E34-BA94-7B2992E7DF23

##### Conservation status

NA

##### Distribution

Calabria, southern Italy

##### Notes

Code: A10; Annex I Birds Directive (2009/147/EC).

#### Charadrius hiaticula

842F6CC9-EABA-51FB-8D5F-BEA83F84922B

##### Distribution

Calabria, southern Italy

##### Notes

Code: A10.

#### Charadrius dubius

3432F6AA-656B-5B52-8606-FD3F456A56BC

##### Conservation status

LC

##### Distribution

Calabria, southern Italy

##### Notes

Code: A11.

#### Charadrius alexandrinus

D6975BC6-56C3-55BB-BA51-5A9004B57772

##### Conservation status

EN

##### Distribution

Calabria, southern Italy

##### Notes

Code: A11; Annex I Birds Directive (2009/147/EC).

#### Vanellus vanellus

0BEC8C36-ED6F-500B-9685-E58E59CE4FEC

##### Conservation status

LC

##### Distribution

Calabria, southern Italy

##### Notes

Code: A10.

#### Vanellus spinosus

10670BFF-AFC7-55B9-84FC-AA5D750BD26A

##### Distribution

Calabria, southern Italy

##### Notes

Code: A30; Annex I Birds Directive (2009/147/EC); Accidental record(s): one record (CS 1986), by Aldo Di Giorgio; the same record is also reported in Scebba et al. (1993).

#### Numenius phaeopus

8904AE18-974F-5B36-9197-95479F44D7B4

##### Distribution

Calabria, southern Italy

##### Notes

Code: A10.

#### Numenius arquata

B37958DB-E077-59EA-8C3D-B3467852AD37

##### Conservation status

NA

##### Distribution

Calabria, southern Italy

##### Notes

Code: A10.

#### Numenius tenuirostris

1EC29117-D29F-5C56-AB31-264743CCFFF4

##### Distribution

Calabria, southern Italy

##### Notes

Code: B40; Annex I Birds Directive (2009/147/EC).

#### Limosa lapponica

AE5FD8E7-2968-5352-BDB0-2F34D1C919C7

##### Distribution

Calabria, southern Italy

##### Notes

Code: A10; Annex I Birds Directive (2009/147/EC).

#### Limosa limosa

074A693F-BDBC-5DB9-9342-B995853A68FB

##### Conservation status

EN

##### Distribution

Calabria, southern Italy

##### Notes

Code: A10.

#### Arenaria interpres

53610F89-B837-5CF1-8943-89EF7E7B3003

##### Distribution

Calabria, southern Italy

##### Notes

Code: A10.

#### Calidris canutus

6BCB17C3-9DD1-5709-8E27-AFA3E29128F2

##### Distribution

Calabria, southern Italy

##### Notes

Code: A10.

#### Calidris pugnax

493FF80D-CB59-5553-859F-FF9D2A92742D

##### Distribution

Calabria, southern Italy

##### Notes

Code: A10; Annex I Birds Directive (2009/147/EC).

#### Calidris falcinellus

0AAA783B-074F-5900-BD84-7035A0020E5C

##### Distribution

Calabria, southern Italy

##### Notes

Code: A30; Accidental record(s): one record (CZ, 22 August 2023), by Miguel Lurgi.

#### Calidris ferruginea

0A1824CD-2350-5794-AD46-FA301E01720D

##### Distribution

Calabria, southern Italy

##### Notes

Code: A10.

#### Calidris temminckii

8AF5D9D6-393F-5D09-9E4F-FE8F6FAC9674

##### Distribution

Calabria, southern Italy

##### Notes

Code: A10.

#### Calidris alba

B57F55BF-A91B-527E-9EE5-F7C5CF7DFF8E

##### Distribution

Calabria, southern Italy

##### Notes

Code: A10.

#### Calidris alpina

5F8656B6-A1E1-510A-9568-AB606FBAA5F2

##### Distribution

Calabria, southern Italy

##### Notes

Code: A10.

#### Calidris maritima

E77E9D6E-24CD-54C6-B9C2-366AFE0B8147

##### Distribution

Calabria, southern Italy

##### Notes

Code: A30; Accidental record(s): one record (KR, 9 August 2018), by Mario Pucci.

#### Calidris minuta

958FF462-4540-5985-9F7D-E3FA6805A214

##### Distribution

Calabria, southern Italy

##### Notes

Code: A10.

#### Calidris melanotos

D5355D7E-A465-5665-8D17-979BB22D55F5

##### Distribution

Calabria, southern Italy

##### Notes

Code: A30; Accidental record(s): three records: (KR, 17 September 2021), by Mario Pucci; (CS, 13 May 2024), by Pierluigi Serravalle; (RC, 26 May 2024), by Carlo Calabrò and Barbara Santostefano.

#### Scolopax rusticola

6FCBD65A-CAD1-5C5F-A8B6-CE9F41169F3B

##### Conservation status

DD

##### Distribution

Calabria, southern Italy

##### Notes

Code: A10.

#### Gallinago media

82CBE5C1-19C5-5771-8E87-53E83F23267C

##### Distribution

Calabria, southern Italy

##### Notes

Code: A10; Annex I Birds Directive (2009/147/EC).

#### Gallinago gallinago

3E529CC6-A226-5131-B775-1FB693752FBB

##### Conservation status

NA

##### Distribution

Calabria, southern Italy

##### Notes

Code: A10.

#### Lymnocryptes minimus

B8A7A089-3E3F-5732-84B6-AD27E03C6866

##### Distribution

Calabria, southern Italy

##### Notes

Code: A10.

#### Phalaropus lobatus

03AF6189-6C81-5322-82C3-A4C351FDAC91

##### Distribution

Calabria, southern Italy

##### Notes

Code: A30; Annex I Birds Directive (2009/147/EC); Accidental record(s): two records: (RC, 11 September 2007), by Giuseppe Martino; (RC, 31 May 2024), by Antonio Aricò.

#### Xenus cinereus

441BF487-5151-5FC3-BC13-D48CEA2F806B

##### Distribution

Calabria, southern Italy

##### Notes

Code: A30; Annex I Birds Directive (2009/147/EC); Accidental record(s): one record (RC 2012), in Martino and Tralongo (2021).

#### Actitis hypoleucos

67A2F3D5-B4F0-5F0D-9DD0-982F4744A969

##### Conservation status

NT

##### Distribution

Calabria, southern Italy

##### Notes

Code: A11.

#### Tringa ochropus

74880311-81BB-55B3-8DE4-1A0626282FB3

##### Distribution

Calabria, southern Italy

##### Notes

Code: A10.

#### Tringa erythropus

386A261E-DDBC-5D08-AF74-8746E32C6570

##### Distribution

Calabria, southern Italy

##### Notes

Code: A10.

#### Tringa nebularia

A1071124-0CC1-5D2D-A703-A36F4CCF19B8

##### Distribution

Calabria, southern Italy

##### Notes

Code: A10.

#### Tringa totanus

7285D4EC-350B-5BB9-8DDE-3577317F7881

##### Conservation status

LC

##### Distribution

Calabria, southern Italy

##### Notes

Code: A10.

#### Tringa glareola

C097A4ED-BE0F-5C99-9D14-C4E295AA0A27

##### Distribution

Calabria, southern Italy

##### Notes

Code: A10; Annex I Birds Directive (2009/147/EC).

#### Tringa stagnatilis

7F09FCDA-FC68-50FD-B760-DC4E2EF2E4C9

##### Distribution

Calabria, southern Italy

##### Notes

Code: A10.

#### Turnix sylvaticus

F248A679-12B0-5A92-9DC0-E0495FC07B84

##### Conservation status

RE

##### Distribution

Calabria, southern Italy

##### Notes

Code: B40; Annex I Birds Directive (2009/147/EC).

#### Cursorius cursor

A41C672D-EB85-5B01-AB0B-B65472660A30

##### Distribution

Calabria, southern Italy

##### Notes

Code: A30; Annex I Birds Directive (2009/147/EC); Accidental record(s): four records: the first two (RC 1898 and KR 1904) reported in Scebba et al. (1993); (RC 1982) by Sergio Tralongo, in Verducci et al. (2012); (RC 2025) by the authors of the present checklist.

#### Glareola pratincola

DF172930-0A1D-5E95-A5E8-491C712DDB96

##### Conservation status

EN

##### Distribution

Calabria, southern Italy

##### Notes

Code: A10; Annex I Birds Directive (2009/147/EC).

#### Hydrocoloeus minutus

06AED6D1-BEB2-51E1-BD7B-3EC9A18CBB6C

##### Distribution

Calabria, southern Italy

##### Notes

Code: A10; Annex I Birds Directive (2009/147/EC).

#### Rissa tridactyla

E01E2EC7-33C1-5876-8ED8-64646744490B

##### Distribution

Calabria, southern Italy

##### Notes

Code: A10.

#### Larus genei

100EBAA3-476D-5B41-B7C2-0C3B8BCDF8D5

##### Conservation status

NT

##### Distribution

Calabria, southern Italy

##### Notes

Code: A10; Annex I Birds Directive (2009/147/EC).

#### Larus ridibundus

60EA9542-DD87-5A7B-A271-BD92F1491D2D

##### Conservation status

LC

##### Distribution

Calabria, southern Italy

##### Notes

Code: A10.

#### Larus melanocephalus

B83B630C-7859-5424-A66D-63530FE428DD

##### Conservation status

NT

##### Distribution

Calabria, southern Italy

##### Notes

Code: A10; Annex I Birds Directive (2009/147/EC).

#### Larus audouinii

DE32E57C-B89D-5170-9163-4111179C92E6

##### Conservation status

LC

##### Distribution

Calabria, southern Italy

##### Notes

Code: A10; Annex I Birds Directive (2009/147/EC).

#### Larus canus

A4E6D97D-8F27-574A-9318-BE5619992F37

##### Distribution

Calabria, southern Italy

##### Notes

Code: A10.

#### Larus fuscus

96D22033-5F85-5540-87D3-BDAA7671E6AD

##### Distribution

Calabria, southern Italy

##### Notes

Code: A10.

#### Larus fuscus fuscus

7F342C97-6784-5F0E-88ED-8B262C55C4D1

##### Distribution

Calabria, southern Italy

##### Notes

Code: A10.

#### Larus michahellis

471BD341-545D-5AEC-8838-0475C3A177F2

##### Conservation status

LC

##### Distribution

Calabria, southern Italy

##### Notes

Code: A11.

#### Larus cachinnans

10FFE675-44EE-5AC2-A5D7-C6FFCB4B85A1

##### Distribution

Calabria, southern Italy

##### Notes

Code: A10.

#### Larus marinus

ED8F47A1-F09A-5F98-A08A-C0E0D51C23CC

##### Distribution

Calabria, southern Italy

##### Notes

Code: A30; Accidental record(s): one record (KR, 13 January 2016), by Eugenio Muscianese.

#### Sternula albifrons

18867616-D46C-5C3B-8B21-30856904B72C

##### Conservation status

NT

##### Distribution

Calabria, southern Italy

##### Notes

Code: A10; Annex I Birds Directive (2009/147/EC).

#### Gelochelidon nilotica

BE2678FB-D164-5FEA-AAA8-10B00AC6B21E

##### Conservation status

NT

##### Distribution

Calabria, southern Italy

##### Notes

Code: A10; Annex I Birds Directive (2009/147/EC).

#### Hydroprogne caspia

1610686D-BB21-5BBE-9823-2856E65903E2

##### Conservation status

NA

##### Distribution

Calabria, southern Italy

##### Notes

Code: A10; Annex I Birds Directive (2009/147/EC).

#### Chlidonias hybrida

9FAD88FA-5F71-56BD-9DA9-C117A2132DA4

##### Conservation status

VU

##### Distribution

Calabria, southern Italy

##### Notes

Code: A10; Annex I Birds Directive (2009/147/EC).

#### Chlidonias leucopterus

722EAFBE-6C73-5403-95CF-BD57CCAD4689

##### Conservation status

NA

##### Distribution

Calabria, southern Italy

##### Notes

Code: A10.

#### Chlidonias niger

81EC7933-B30D-50FF-AED9-552D1B353408

##### Conservation status

CR

##### Distribution

Calabria, southern Italy

##### Notes

Code: A10; Annex I Birds Directive (2009/147/EC).

#### Sterna hirundo

CFCE5B68-76E6-5B84-AAF6-1687631E2DCD

##### Conservation status

LC

##### Distribution

Calabria, southern Italy

##### Notes

Code: A10; Annex I Birds Directive (2009/147/EC).

#### Thalasseus sandvicensis

22793A47-805F-5049-B36F-C12A2E97C205

##### Conservation status

VU

##### Distribution

Calabria, southern Italy

##### Notes

Code: A10; Annex I Birds Directive (2009/147/EC).

#### Stercorarius parasiticus

372E6E9F-1D46-5B93-8B8A-560902C4AB74

##### Distribution

Calabria, southern Italy

##### Notes

Code: A10.

#### Stercorarius pomarinus

7FC5D958-1D65-5137-929F-4A02FDD0255B

##### Distribution

Calabria, southern Italy

##### Notes

Code: A10.

#### Catharacta skua

C2265875-CC58-54DD-970C-C80A9545D8EF

##### Distribution

Calabria, southern Italy

##### Notes

Code: A30; Accidental record(s): one record (CS, 25 August 2006), two individuals observed by Davide De Rosa.

#### Fratercula arctica

9BDD1EEB-68C4-51D7-A192-63869F348EF1

##### Distribution

Calabria, southern Italy

##### Notes

Code: A30; Accidental record(s): six records (date and locality unknown) in Scebba et al. (1993).

#### Alca torda

51161DA0-A49F-56FF-AA33-762C8DF1ACF3

##### Distribution

Calabria, southern Italy

##### Notes

Code: A20.

#### Tyto alba

5F4A26DC-E57E-5C9A-A771-6D760C82AA82

##### Conservation status

LC

##### Distribution

Calabria, southern Italy

##### Notes

Code: A11.

#### Athene noctua

D5790108-4B2A-59DD-8749-36AEF546F8F3

##### Conservation status

LC

##### Distribution

Calabria, southern Italy

##### Notes

Code: A11.

#### Otus scops

52237E0A-9C0A-5479-8CC9-12120BAC927A

##### Conservation status

LC

##### Distribution

Calabria, southern Italy

##### Notes

Code: A11.

#### Asio otus

453B629E-5F14-596D-A965-0A6C1AB5E99F

##### Conservation status

LC

##### Distribution

Calabria, southern Italy

##### Notes

Code: A11.

#### Asio flammeus

FBE8CCA0-2B80-540F-A725-7480826683DF

##### Conservation status

NA

##### Distribution

Calabria, southern Italy

##### Notes

Code: A10; Annex I Birds Directive (2009/147/EC).

#### Strix aluco

BC9C8B1D-47D2-5F18-A794-1F9D4D0D3451

##### Conservation status

LC

##### Distribution

Calabria, southern Italy

##### Notes

Code: A11.

#### Bubo bubo

37E457A9-9088-5655-9A06-E251FD769721

##### Conservation status

NT

##### Distribution

Calabria, southern Italy

##### Notes

Code: A11; Annex I Birds Directive (2009/147/EC).

#### Pandion haliaetus

2D497433-F310-5CB7-86F8-A9CAEEAE1311

##### Conservation status

CR

##### Distribution

Calabria, southern Italy

##### Notes

Code: A10; Annex I Birds Directive (2009/147/EC).

#### Elanus caeruleus

1117FE57-27A6-5EDA-B295-09793ACAAC5E

##### Distribution

Calabria, southern Italy

##### Notes

Code: A30; Annex I Birds Directive (2009/147/EC); Accidental record(s): four records: (CS 1969 and CZ 1974) reported in Scebba et al. (1993); (RC, 29 September 2010) by Giuseppe Martino and Elena Grasso; (KR, 25 March 2019) by Mario Pucci.

#### Pernis apivorus

0F1478AF-1464-5BC7-A70B-BC4A6E5490F5

##### Conservation status

LC

##### Distribution

Calabria, southern Italy

##### Notes

Code: A11; Annex I Birds Directive (2009/147/EC).

#### Pernis ptilorhynchus

48F6DA26-48F2-5DAE-AF53-105DB904DD48

##### Distribution

Calabria, southern Italy

##### Notes

Code: A30; Accidental record(s): one record (RC 2011), in Scuderi and Corso (2011).

#### Gypaetus barbatus

C9E8114E-7892-5CD0-ADDA-11E9730836EC

##### Conservation status

CR

##### Distribution

Calabria, southern Italy

##### Notes

Code: A34; Annex I Birds Directive (2009/147/EC).

#### Neophron percnopterus

4948225E-13CB-5431-94C9-D50D37DD8D14

##### Conservation status

CR

##### Distribution

Calabria, southern Italy

##### Notes

Code: A11; Annex I Birds Directive (2009/147/EC).

#### Circaetus gallicus

FDE99321-8ABC-5F68-8F7B-65B6095CD974

##### Conservation status

LC

##### Distribution

Calabria, southern Italy

##### Notes

Code: A11; Annex I Birds Directive (2009/147/EC).

#### Gyps rueppellii

AAD035F8-15AB-5C65-AFFE-32BFADCD1D43

##### Distribution

Calabria, southern Italy

##### Notes

Code: A30; Accidental record(s): two records: (CS, 25 May 2011) one individual photographed by Gianni De Marco at Civita (CS), on the acclimatisation aviaries for Griffon Vultures; (CS, 23 November 2023) by Pierluigi Serravalle.

#### Gyps fulvus

E0764442-EB35-5338-9153-2E35901D4E4F

##### Conservation status

NT

##### Distribution

Calabria, southern Italy

##### Notes

Code: AC12; Annex I Birds Directive (2009/147/EC).

#### Aegypius monachus

7E28CCF5-9A13-58B0-8909-06CF5699C67D

##### Conservation status

RE

##### Distribution

Calabria, southern Italy

##### Notes

Code: A34; Annex I Birds Directive (2009/147/EC).

#### Clanga pomarina

B3F56EF4-F4C1-5E00-91E0-2A20DBF0A631

##### Distribution

Calabria, southern Italy

##### Notes

Code: A10; Annex I Birds Directive (2009/147/EC).

#### Clanga clanga

CA41A4BA-B7A2-5970-92F6-AE338B80A3AA

##### Distribution

Calabria, southern Italy

##### Notes

Code: A20; Annex I Birds Directive (2009/147/EC).

#### Aquila nipalensis

DCCFF03D-66A3-5948-A9A6-2F912C5EE56D

##### Distribution

Calabria, southern Italy

##### Notes

Code: A30; Accidental record(s): two records: (RC, 22 March 2022), by Michele Cento; (KR, 2 May 2022), in Muscianese et al. (2023).

#### Aquila heliaca

CF01E215-10BF-5F73-9BC4-364B3400AF04

##### Distribution

Calabria, southern Italy

##### Notes

Code: A20; Annex I Birds Directive (2009/147/EC).

#### Aquila chrysaetos

B422BEAD-1F94-5080-A6C9-5D43DFDFD274

##### Conservation status

NT

##### Distribution

Calabria, southern Italy

##### Notes

Code: A11; Annex I Birds Directive (2009/147/EC).

#### Aquila fasciata

3EC36E95-A0E4-5E46-A06F-52C4489AE9D2

##### Conservation status

EN

##### Distribution

Calabria, southern Italy

##### Notes

Code: A20; Annex I Birds Directive (2009/147/EC).

#### Hieraaetus pennatus

548B8E2A-A1EA-50AC-87E6-C3C56D9D1759

##### Conservation status

NA

##### Distribution

Calabria, southern Italy

##### Notes

Code: A10; Annex I Birds Directive (2009/147/EC).

#### Circus aeruginosus

AE0399F9-BA4B-54FD-B36C-DBE09D124ECC

##### Conservation status

VU

##### Distribution

Calabria, southern Italy

##### Notes

Code: A10; Annex I Birds Directive (2009/147/EC).

#### Circus cyaneus

AA9252E4-0BA1-5148-B4D9-6596C8F7497B

##### Conservation status

NA

##### Distribution

Calabria, southern Italy

##### Notes

Code: A11; Annex I Birds Directive (2009/147/EC).

#### Circus macrourus

353916C9-8498-59D3-B705-597C20A07B07

##### Distribution

Calabria, southern Italy

##### Notes

Code: A10; Annex I Birds Directive (2009/147/EC).

#### Circus pygargus

5A06845C-B3C6-5786-A68A-D428AA348B95

##### Conservation status

VU

##### Distribution

Calabria, southern Italy

##### Notes

Code: A10; Annex I Birds Directive (2009/147/EC).

#### Accipiter brevipes

AD6C0FC6-82FC-518C-868F-F01AE3002BD6

##### Distribution

Calabria, southern Italy

##### Notes

Code: B40; Annex I Birds Directive (2009/147/EC).

#### Accipiter nisus

F7654273-BD13-52B1-A5BC-65F8AD30B38C

##### Conservation status

LC

##### Distribution

Calabria, southern Italy

##### Notes

Code: A11.

#### Accipiter gentilis

B767C4FF-AD33-5352-9B11-5D9EC4F9B91D

##### Conservation status

LC

##### Distribution

Calabria, southern Italy

##### Notes

Code: A11.

#### Haliaeetus albicilla

F928D943-51E8-5A3E-AD78-75B87A481B90

##### Distribution

Calabria, southern Italy

##### Notes

Code: A30; Annex I Birds Directive (2009/147/EC); Accidental record(s): three records (RC 1973, 1974 and 1975), in Scebba et al. (1993).

#### Milvus milvus

CA26A0EF-8C03-5D2C-89EA-FF049918E88C

##### Conservation status

VU

##### Distribution

Calabria, southern Italy

##### Notes

Code: A11; Annex I Birds Directive (2009/147/EC).

#### Milvus migrans

AC1297D6-2D66-504F-9E46-E69753144E4A

##### Conservation status

LC

##### Distribution

Calabria, southern Italy

##### Notes

Code: A11; Annex I Birds Directive (2009/147/EC).

#### Buteo lagopus

93D2CC5A-47A3-5BEA-9306-F295013FAC61

##### Distribution

Calabria, southern Italy

##### Notes

Code: A30; Accidental record(s): one record (CS 1972), in Scebba et al. (1993).

#### Buteo buteo

61B21CDB-5364-566F-BEB7-84A0A4392F9F

##### Conservation status

LC

##### Distribution

Calabria, southern Italy

##### Notes

Code: A11.

#### Buteo buteo vulpinus

0EA03090-57EB-5AA6-BC34-F8F2C76A4380

##### Distribution

Calabria, southern Italy

##### Notes

Code: A10.

#### Buteo rufinus

D9AB643B-5D70-55D2-8761-DFAA86639B7B

##### Distribution

Calabria, southern Italy

##### Notes

Code: A10; Annex I Birds Directive (2009/147/EC).

#### Upupa epops

72E7D395-EE35-5027-AF9C-B7D4A6947A96

##### Conservation status

LC

##### Distribution

Calabria, southern Italy

##### Notes

Code: A11.

#### Merops persicus

9E03DD49-9A92-5516-8BA0-4F71093646B3

##### Distribution

Calabria, southern Italy

##### Notes

Code: A30; Accidental record(s): two records: (RC, 8 May 2009), by Giuseppe Martino; (RC, 20 April 2025), by Michele Cento.

#### Merops apiaster

61C7B413-076C-5917-974F-4A075D3CB8BE

##### Conservation status

LC

##### Distribution

Calabria, southern Italy

##### Notes

Code: A11.

#### Coracias garrulus

A40AB7C5-E0DB-506A-9D23-F588A8685C6D

##### Conservation status

LC

##### Distribution

Calabria, southern Italy

##### Notes

Code: A11; Annex I Birds Directive (2009/147/EC).

#### Alcedo atthis

848D56E6-684B-5A03-9E6F-B1D3B9A51E4E

##### Conservation status

NT

##### Distribution

Calabria, southern Italy

##### Notes

Code: A11; Annex I Birds Directive (2009/147/EC).

#### Jynx torquilla

6A4C0C09-F86A-5761-BF5F-19567AA9AE76

##### Conservation status

EN

##### Distribution

Calabria, southern Italy

##### Notes

Code: A11.

#### Picus viridis

C151DB07-BE47-582D-BF6A-CBE42B10FA39

##### Conservation status

LC

##### Distribution

Calabria, southern Italy

##### Notes

Code: A11.

#### Dryocopus martius

482EC62A-8097-5DDC-B20E-688E164A6D44

##### Conservation status

LC

##### Distribution

Calabria, southern Italy

##### Notes

Code: A11; Annex I Birds Directive (2009/147/EC).

#### Leiopicus medius

CE5B4816-A900-573B-A2C0-C95BAEB0365C

##### Conservation status

VU

##### Distribution

Calabria, southern Italy

##### Notes

Code: A11; Annex I Birds Directive (2009/147/EC).

#### Dryobates minor

5B234858-0312-5004-B6F0-1F6CCC22CE9B

##### Conservation status

LC

##### Distribution

Calabria, southern Italy

##### Notes

Code: A11.

#### Dendrocopos major

989E8975-FFD1-5E9E-AEA3-1E24C37AE498

##### Conservation status

LC

##### Distribution

Calabria, southern Italy

##### Notes

Code: A11.

#### Falco naumanni

C4189202-2E4D-5B14-95C2-1C858B65E0F5

##### Conservation status

LC

##### Distribution

Calabria, southern Italy

##### Notes

Code: A11; Annex I Birds Directive (2009/147/EC).

#### Falco tinnunculus

696F7767-59D4-5E0E-AA29-71F973B467D0

##### Conservation status

LC

##### Distribution

Calabria, southern Italy

##### Notes

Code: A11.

#### Falco vespertinus

0AA769BB-D974-5CEB-AE33-A9322DD8C382

##### Conservation status

VU

##### Distribution

Calabria, southern Italy

##### Notes

Code: A10; Annex I Birds Directive (2009/147/EC).

#### Falco eleonorae

37B7596E-597A-58B5-B24B-FDB76E9BBCCC

##### Conservation status

VU

##### Distribution

Calabria, southern Italy

##### Notes

Code: A10; Annex I Birds Directive (2009/147/EC).

#### Falco columbarius

74B5DA1F-C9D5-5168-ACAD-567B8233EF7D

##### Distribution

Calabria, southern Italy

##### Notes

Code: A10; Annex I Birds Directive (2009/147/EC).

#### Falco subbuteo

F9431BE1-3CDD-5E25-8A80-D5BB6DD3BD18

##### Conservation status

LC

##### Distribution

Calabria, southern Italy

##### Notes

Code: A11.

#### Falco biarmicus

0E25A2CA-A065-5EAB-96C2-FDDFC58A028A

##### Conservation status

EN

##### Distribution

Calabria, southern Italy

##### Notes

Code: A11; Annex I Birds Directive (2009/147/EC).

#### Falco cherrug

E8C6E9DB-9AB4-5167-9232-FABE7D8C555C

##### Distribution

Calabria, southern Italy

##### Notes

Code: A10; Annex I Birds Directive (2009/147/EC).

#### Falco peregrinus

65CDCBF4-1A23-5062-9636-CBB5B2D98129

##### Conservation status

LC

##### Distribution

Calabria, southern Italy

##### Notes

Code: A11; Annex I Birds Directive (2009/147/EC).

#### Falco peregrinus calidus

447142E4-3547-561A-889E-1F76F76D69D3

##### Distribution

Calabria, southern Italy

##### Notes

Code: A20.

#### Psittacula krameri

062A35B8-A93C-5E52-B604-6BBB21DD6EE5

##### Distribution

Calabria, southern Italy

##### Notes

Code: C22.

#### Oriolus oriolus

F200F685-43CA-5B91-9D02-FE8F4878A737

##### Conservation status

LC

##### Distribution

Calabria, southern Italy

##### Notes

Code: A11.

#### Lanius cristatus

8917CAC3-B269-5CC5-A7BF-EF276A946FC8

##### Distribution

Calabria, southern Italy

##### Notes

Code: A30; Accidental record(s): one record (KR, 1 May 2022), by Mario Pucci.

#### Lanius collurio

39C31D21-2F0B-54F5-A9BD-35E0D875476F

##### Conservation status

VU

##### Distribution

Calabria, southern Italy

##### Notes

Code: A11; Annex I Birds Directive (2009/147/EC).

#### Lanius isabellinus

44EC25D8-D072-5462-923B-CCB1E9659FD6

##### Distribution

Calabria, southern Italy

##### Notes

Code: A30; Accidental record(s): one record (RC, 29 November 2016), in Martino and Tralongo (2021).

#### Lanius minor

002C109B-791B-56B9-8B22-3A39A3B41A99

##### Conservation status

EN

##### Distribution

Calabria, southern Italy

##### Notes

Code: A11; Annex I Birds Directive (2009/147/EC).

#### Lanius excubitor

F4D88F5B-FF04-57B0-AD6F-81013F4E9A87

##### Distribution

Calabria, southern Italy

##### Notes

Code: A30; Accidental record(s): three records: (RC 1975) in Scebba et al. (1993); (RC, 19 March 2017), by Manuela Policastrese; (CS, 30 November 2025), by Pierluigi Serravalle.

#### Lanius excubitor pallidirostris

B6BC270B-165B-527D-8DB4-ADF565B92C1A

##### Distribution

Calabria, southern Italy

##### Notes

Code: A30; Accidental record(s): two records: (RC, 22 December 2016), in Martino and Tralongo (2021); (KR, 2 December 2022), by Mario Pucci.

#### Lanius senator

682F1716-7182-50CA-8E4E-22768E3E925B

##### Conservation status

EN

##### Distribution

Calabria, southern Italy

##### Notes

Code: A11.

#### Lanius nubicus

6EAAE509-9403-510D-82B4-06D568397813

##### Distribution

Calabria, southern Italy

##### Notes

Code: A30; Annex I Birds Directive (2009/147/EC); Accidental record(s): one record (RC 2017), by Mario Pucci in Fracasso et al. (2018).

#### Pyrrhocorax pyrrhocorax

C94B41FE-8838-56AD-A4B3-E42906AABF4A

##### Conservation status

LC

##### Distribution

Calabria, southern Italy

##### Notes

Code: A40.

#### Garrulus glandarius

6D7C5609-D8B9-55BD-AE9C-007AB345FBFB

##### Conservation status

LC

##### Distribution

Calabria, southern Italy

##### Notes

Code: A11.

#### Pica pica

0F9D2552-F56D-5A79-977C-C05EA025B07F

##### Conservation status

LC

##### Distribution

Calabria, southern Italy

##### Notes

Code: A11.

#### Corvus monedula

5172F8A1-60EA-5ED4-A61F-A8E4D709E17E

##### Conservation status

LC

##### Distribution

Calabria, southern Italy

##### Notes

Code: A11.

#### Corvus frugilegus

2B1F486D-FA56-550B-AC95-573D446D1FD8

##### Distribution

Calabria, southern Italy

##### Notes

Code: A30; Accidental record(s): three records: (RC, 10 May 2010), by Michele Cento; (RC, 16 March 2018), by Simonetta Cutini; (RC, 14 June 2019), by Domenico Bevacqua.

#### Corvus corax

47D4C548-6273-57F0-BE8E-1D3149ADC188

##### Conservation status

LC

##### Distribution

Calabria, southern Italy

##### Notes

Code: A11.

#### Corvus corone

E096106B-FAE5-597F-B28D-2CD839C2960D

##### Conservation status

LC

##### Distribution

Calabria, southern Italy

##### Notes

Code: A11.

#### Periparus ater

D27D7808-12CC-5592-99FB-998AF5A022A4

##### Conservation status

LC

##### Distribution

Calabria, southern Italy

##### Notes

Code: A11.

#### Poecile palustris

819CD5CD-DB27-5F53-A80E-2DF8E312CC0B

##### Conservation status

LC

##### Distribution

Calabria, southern Italy

##### Notes

Code: A11.

#### Cyanistes caeruleus

97F1AC50-DEE7-5B9C-847D-F51334DFD8BE

##### Conservation status

LC

##### Distribution

Calabria, southern Italy

##### Notes

Code: A11.

#### Parus major

0B93EDE5-9999-52CF-B659-01D7D233DA94

##### Conservation status

LC

##### Distribution

Calabria, southern Italy

##### Notes

Code: A11.

#### Remiz pendulinus

1B090797-FF46-5930-B6E8-967C52404CAB

##### Conservation status

VU

##### Distribution

Calabria, southern Italy

##### Notes

Code: A11.

#### Alaudala rufescens

97D3C085-8A26-500C-9DD8-93401DF5E8F4

##### Distribution

Calabria, southern Italy

##### Notes

Code: A30; Accidental record(s): one record (KR, 21 April 2025), by Mario Pucci.

#### Melanocorypha calandra

DADD6CDA-5DB2-5B1C-9EB4-7CE6F1BC8F9F

##### Conservation status

VU

##### Distribution

Calabria, southern Italy

##### Notes

Code: A11; Annex I Birds Directive (2009/147/EC).

#### Calandrella brachydactyla

AA5FBBAA-00EB-554C-AF09-D98250E8D66E

##### Conservation status

LC

##### Distribution

Calabria, southern Italy

##### Notes

Code: A11; Annex I Birds Directive (2009/147/EC).

#### Eremophila alpestris

364761B6-7050-5010-9725-EB84625EE53A

##### Distribution

Calabria, southern Italy

##### Notes

Code: A30; Accidental record(s): three records: two historical records (pre-1950; RC 1898 or 1899) and one more recent (CS 1982), all reported in Scebba et al. (1993).

#### Lullula arborea

34D88DAD-DD38-53E1-B66C-A52D739DF340

##### Conservation status

LC

##### Distribution

Calabria, southern Italy

##### Notes

Code: A11; Annex I Birds Directive (2009/147/EC).

#### Alauda arvensis

FB66493F-C9E8-5E06-95AD-7D30EE6D44BF

##### Conservation status

VU

##### Distribution

Calabria, southern Italy

##### Notes

Code: A11.

#### Galerida cristata

08F452E3-FFBC-533E-A62C-3DBA747E1778

##### Conservation status

LC

##### Distribution

Calabria, southern Italy

##### Notes

Code: A11.

#### Panurus biarmicus

64F07B8C-21F1-5D78-A945-7AE14B0769CD

##### Conservation status

EN

##### Distribution

Calabria, southern Italy

##### Notes

Code: B40.

#### Cisticola juncidis

E4F10FC3-0EF4-5F2F-B779-70F841B752BA

##### Conservation status

LC

##### Distribution

Calabria, southern Italy

##### Notes

Code: A11.

#### Hippolais polyglotta

9D1D1DD3-8F59-52B7-B548-202D48CEB2BF

##### Conservation status

LC

##### Distribution

Calabria, southern Italy

##### Notes

Code: A11.

#### Hippolais icterina

8AEC8ACA-8D4A-531B-AEA5-F395532F6A6E

##### Distribution

Calabria, southern Italy

##### Notes

Code: A10.

#### Acrocephalus melanopogon

E6747772-42FF-5BCB-BB80-C9BF034BB824

##### Conservation status

EN

##### Distribution

Calabria, southern Italy

##### Notes

Code: A10; Annex I Birds Directive (2009/147/EC).

#### Acrocephalus schoenobaenus

0B60762C-9210-57E1-8612-651D67345577

##### Conservation status

CR

##### Distribution

Calabria, southern Italy

##### Notes

Code: A10.

#### Acrocephalus palustris

1992F5D0-B6F6-581F-A7F5-3F4C6D43CE9B

##### Conservation status

NT

##### Distribution

Calabria, southern Italy

##### Notes

Code: A20.

#### Acrocephalus scirpaceus

59C1C6AA-FA0F-5863-962A-9C11BB200AA8

##### Conservation status

LC

##### Distribution

Calabria, southern Italy

##### Notes

Code: A11.

#### Acrocephalus arundinaceus

77B3CA89-EADC-5A69-9645-2457D171C44A

##### Conservation status

NT

##### Distribution

Calabria, southern Italy

##### Notes

Code: A11.

#### Locustella luscinioides

D849F541-2A91-5F5F-BC9C-BAA552424F6D

##### Conservation status

EN

##### Distribution

Calabria, southern Italy

##### Notes

Code: A20.

#### Delichon urbicum

D0DE672D-18D6-56E1-A855-77E0218D9358

##### Conservation status

NT

##### Distribution

Calabria, southern Italy

##### Notes

Code: A11.

#### Cecropis daurica

F41061CF-D45E-51C3-A680-35F33C191A77

##### Conservation status

VU

##### Distribution

Calabria, southern Italy

##### Notes

Code: A11.

#### Hirundo rustica

992E8DF9-C68E-579A-9DCD-6DC4BEAA6967

##### Conservation status

NT

##### Distribution

Calabria, southern Italy

##### Notes

Code: A11.

#### Ptyonoprogne rupestris

1577B35D-275D-532C-879C-27E080A31DE6

##### Conservation status

LC

##### Distribution

Calabria, southern Italy

##### Notes

Code: A11.

#### Riparia riparia

31C58FD4-7929-5567-81F4-72B8510C8A1A

##### Conservation status

VU

##### Distribution

Calabria, southern Italy

##### Notes

Code: A13.

#### Phylloscopus bonelli

701AF9C6-2837-5C10-B9F0-4824C5489D4C

##### Conservation status

LC

##### Distribution

Calabria, southern Italy

##### Notes

Code: A11.

#### Phylloscopus sibilatrix

8B339EA4-BCDE-5A8E-ADD8-CEE32C35B5CB

##### Conservation status

LC

##### Distribution

Calabria, southern Italy

##### Notes

Code: A11.

#### Phylloscopus inornatus

DDB62111-D10E-567B-B0D0-CEC7A3B94371

##### Distribution

Calabria, southern Italy

##### Notes

Code: A30; Accidental record(s): three records referring to three different individuals ringed in 2020 by Mario Pucci at the Punta Alice ringing station (KR).

#### Phylloscopus proregulus

C07104AF-E4CC-550D-93D1-1279D1B294A2

##### Distribution

Calabria, southern Italy

##### Notes

Code: A30; Accidental record(s): one record (CS 2005), in Policastrese et al. (2006).

#### Phylloscopus fuscatus

B83E00CC-1105-52D0-B013-069F0C9AECF8

##### Distribution

Calabria, southern Italy

##### Notes

Code: A30; Accidental record(s): one record referring to one individual ringed in 2017 by Mario Pucci at the Punta Alice ringing station (KR).

#### Phylloscopus trochilus

763D7325-ADC9-5A39-945B-8970D50A5321

##### Distribution

Calabria, southern Italy

##### Notes

Code: A10.

#### Phylloscopus collybita

3D5CFCE2-1C2B-5FDD-97E6-682AFD085582

##### Conservation status

LC

##### Distribution

Calabria, southern Italy

##### Notes

Code: A11.

#### Phylloscopus tristis

CA29C4B8-057F-54A6-81EA-92E0AFDE4F9E

##### Distribution

Calabria, southern Italy

##### Notes

Code: A20.

#### Cettia cetti

D956B475-125A-58C9-A3F7-FC7A3F3D5A89

##### Conservation status

LC

##### Distribution

Calabria, southern Italy

##### Notes

Code: A11.

#### Aegithalos caudatus

AED7CC30-0949-58EF-94C8-13E8D4634F8C

##### Conservation status

LC

##### Distribution

Calabria, southern Italy

##### Notes

Code: A11.

#### Sylvia atricapilla

1DF0F7A2-B29D-566D-BD5B-01B566004B8F

##### Conservation status

LC

##### Distribution

Calabria, southern Italy

##### Notes

Code: A11.

#### Sylvia borin

71DAE541-5714-5BDE-8EB9-C945EA5B43F2

##### Conservation status

EN

##### Distribution

Calabria, southern Italy

##### Notes

Code: A10.

#### Sylvia nisoria

BC3AFF65-E58E-5F27-AE01-886AF10CAC00

##### Conservation status

CR

##### Distribution

Calabria, southern Italy

##### Notes

Code: B40; Annex I Birds Directive (2009/147/EC).

#### Sylvia hortensis

689545F0-987D-5F49-8FB5-4939DD0E6215

##### Conservation status

EN

##### Distribution

Calabria, southern Italy

##### Notes

Code: B40.

#### Sylvia curruca

AB168D45-FB17-53D5-9CE3-D91BD0665B35

##### Conservation status

LC

##### Distribution

Calabria, southern Italy

##### Notes

Code: A10.

#### Sylvia melanocephala

92D0978E-079B-5A2A-BDB2-1226623E03A1

##### Conservation status

LC

##### Distribution

Calabria, southern Italy

##### Notes

Code: A11.

#### Sylvia cantillans

2FCE43F7-A4C1-52D7-BA21-D7DDA0FB398A

##### Conservation status

LC

##### Distribution

Calabria, southern Italy

##### Notes

Code: A11.

#### Sylvia ruppeli

53E49BD2-6359-5EB3-A557-0E6C55C4327A

##### Distribution

Calabria, southern Italy

##### Notes

Code: A30; Annex I Birds Directive (2009/147/EC); Accidental record(s): seven records referring to seven different individuals ringed in 2016, 2019, 2020, 2022 and 2025 by Mario Pucci at the Punta Alice ringing station (KR).

#### Sylvia communis

325D89EB-91A1-5626-93F3-0E6E94B12541

##### Conservation status

LC

##### Distribution

Calabria, southern Italy

##### Notes

Code: A11.

#### Sylvia conspicillata

1E63E3B0-0FA3-58B4-8B6E-2CA0F355332B

##### Conservation status

LC

##### Distribution

Calabria, southern Italy

##### Notes

Code: A11.

#### Sylvia sarda

95E6CF1A-10C4-548B-A2CD-B21D05FA107D

##### Conservation status

DD

##### Distribution

Calabria, southern Italy

##### Notes

Code: A30; Annex I Birds Directive (2009/147/EC); Accidental record(s): one record (date and locality unknown), in Scebba et al. (1993).

#### Sylvia undata

F658FA4F-7A5A-5175-8908-ECE79D3F615D

##### Conservation status

DD

##### Distribution

Calabria, southern Italy

##### Notes

Code: A11; Annex I Birds Directive (2009/147/EC).

#### Certhia brachydactyla

A1F05E45-0D04-5B12-B33C-DB9727A04227

##### Conservation status

LC

##### Distribution

Calabria, southern Italy

##### Notes

Code: A11.

#### Certhia familiaris

8DA452C5-8AD1-528D-9C30-183BA85763ED

##### Conservation status

LC

##### Distribution

Calabria, southern Italy

##### Notes

Code: A11.

#### Sitta europaea

96032461-04C6-5CDB-BF29-7E40B3F41A41

##### Conservation status

LC

##### Distribution

Calabria, southern Italy

##### Notes

Code: A11.

#### Tichodroma muraria

4AF2B03A-231D-5A5D-B7CB-6F9FC6DC13BD

##### Conservation status

LC

##### Distribution

Calabria, southern Italy

##### Notes

Code: A10.

#### Troglodytes troglodytes

E3195672-9124-5120-9212-DAD55DF3BBBE

##### Conservation status

LC

##### Distribution

Calabria, southern Italy

##### Notes

Code: A11.

#### Cinclus cinclus

CA16431B-2DC7-5F9A-9C71-1179D2CC3E54

##### Conservation status

LC

##### Distribution

Calabria, southern Italy

##### Notes

Code: A11.

#### Sturnus vulgaris

37358DD5-1366-524E-A7F8-AB4211E55362

##### Conservation status

LC

##### Distribution

Calabria, southern Italy

##### Notes

Code: A11.

#### Pastor roseus

0B5A5ABB-C2CE-5AF7-819E-C49A7515F3EE

##### Distribution

Calabria, southern Italy

##### Notes

Code: A30; Accidental record(s): five records: (KR 1883 and RC 1888) in Scebba et al. (1993); (RC, 13 August 2009) and (KR, 31 May 2021 [2]) reported, respectively, by Giuseppe Martino and Mario Pucci.

#### Turdus viscivorus

A6FE56D2-F8B1-56FA-BC9B-41F371036357

##### Conservation status

LC

##### Distribution

Calabria, southern Italy

##### Notes

Code: A11.

#### Turdus philomelos

A249C4FE-AB03-5FE3-9BCA-5414EF107365

##### Conservation status

LC

##### Distribution

Calabria, southern Italy

##### Notes

Code: A11.

#### Turdus iliacus

0317CFAF-0D4C-5624-BF1A-6E936D6B569B

##### Conservation status

NA

##### Distribution

Calabria, southern Italy

##### Notes

Code: A10.

#### Turdus merula

0ABBA8EF-280D-5F26-B3F7-B2604CDBE86C

##### Conservation status

LC

##### Distribution

Calabria, southern Italy

##### Notes

Code: A11.

#### Turdus obscurus

724074EB-9E78-5221-B766-B38ECB9A7944

##### Distribution

Calabria, southern Italy

##### Notes

Code: A30; Accidental record(s): four records: (RC 2012, RC 2015, RC 2020, VV 2023) by Giuseppe Martino, all resulting from hunting activity.

#### Turdus pilaris

7680416D-2876-5EA2-911A-F505EC568937

##### Conservation status

VU

##### Distribution

Calabria, southern Italy

##### Notes

Code: A10.

#### Turdus torquatus

AEFC5888-641C-54F5-A902-AC64BE71CA83

##### Conservation status

LC

##### Distribution

Calabria, southern Italy

##### Notes

Code: A20.

#### Turdus torquatus alpestris

168B343F-8FBC-5ECD-9C06-A5B79A2721C3

##### Distribution

Calabria, southern Italy

##### Notes

Code: A30; Accidental record(s): one record (RC, 20 October 2021), by Giuseppe Martino.

#### Muscicapa striata

85F46294-9B6C-597A-BBC4-675CB9D5A137

##### Conservation status

LC

##### Distribution

Calabria, southern Italy

##### Notes

Code: A11.

#### Erithacus rubecula

AB61D653-C50F-5D27-86AA-78FB37E29DFB

##### Conservation status

LC

##### Distribution

Calabria, southern Italy

##### Notes

Code: A11.

#### Cyanecula svecica

E25FD856-6FDA-5FE2-87C7-F43D0A127F97

##### Conservation status

NA

##### Distribution

Calabria, southern Italy

##### Notes

Code: A20; Annex I Birds Directive (2009/147/EC).

#### Luscinia megarhynchos

D145DD8A-CECF-50D7-B2C6-383A07D59AE1

##### Conservation status

LC

##### Distribution

Calabria, southern Italy

##### Notes

Code: A11.

#### Calliope calliope

D2613BF4-1BFE-515F-80E3-E52779B94CA1

##### Distribution

Calabria, southern Italy

##### Notes

Code: A40.

#### Tarsiger cyanurus

72BA9E6A-EE50-5F62-9066-22D7F47BEAF2

##### Distribution

Calabria, southern Italy

##### Notes

Code: A30; Accidental record(s): one record (CS, 15 January 2012), by Maurizio Vena and Salvatore Urso.

#### Ficedula parva

DDC4450C-5D97-5941-B262-6B0450409DBE

##### Distribution

Calabria, southern Italy

##### Notes

Code: A30; Annex I Birds Directive (2009/147/EC); Accidental record(s): one record (CZ, 20 October 2007), by Domenico Bevacqua, during ringing activity at the mouth of the Crocchio River.

#### Ficedula hypoleuca

A39EAC77-75CD-5526-B892-3B72E75543C2

##### Conservation status

NA

##### Distribution

Calabria, southern Italy

##### Notes

Code: A10.

#### Ficedula albicollis

24855F74-51BB-5E47-9693-AEDF160168A6

##### Conservation status

LC

##### Distribution

Calabria, southern Italy

##### Notes

Code: A11; Annex I Birds Directive (2009/147/EC).

#### Phoenicurus ochruros

617F6EF8-62F8-5442-B0D7-8BBF0DDDAA45

##### Conservation status

LC

##### Distribution

Calabria, southern Italy

##### Notes

Code: A11.

#### Phoenicurus phoenicurus

1C52678F-465C-5BB6-A265-A2F526ECA439

##### Conservation status

LC

##### Distribution

Calabria, southern Italy

##### Notes

Code: A11.

#### Phoenicurus moussieri

192DE735-A3D7-5CFA-ABFD-7A1FA421F456

##### Distribution

Calabria, southern Italy

##### Notes

Code: B40.

#### Monticola saxatilis

E8CEA87D-AD7B-579C-AF8F-E1DE54BE5B8C

##### Conservation status

DD

##### Distribution

Calabria, southern Italy

##### Notes

Code: A11.

#### Monticola solitarius

2F99D849-04B3-5F25-9D76-0701259FDFA7

##### Conservation status

NT

##### Distribution

Calabria, southern Italy

##### Notes

Code: A11.

#### Saxicola rubetra

1555A279-9F97-56E1-88B4-24C689429446

##### Conservation status

VU

##### Distribution

Calabria, southern Italy

##### Notes

Code: A11.

#### Saxicola torquatus

017E024E-1209-597C-A797-41A4264ACD27

##### Conservation status

EN

##### Distribution

Calabria, southern Italy

##### Notes

Code: A11.

#### Oenanthe oenanthe

6F765973-4113-513E-A37F-1B43DABC909C

##### Conservation status

LC

##### Distribution

Calabria, southern Italy

##### Notes

Code: A11.

#### Oenanthe isabellina

566AE74C-A223-5B33-AAD0-58414C5A6BFC

##### Distribution

Calabria, southern Italy

##### Notes

Code: A10.

#### Oenanthe deserti

366CD57E-55BA-5895-94D8-B6D7AE269187

##### Distribution

Calabria, southern Italy

##### Notes

Code: A30; Accidental record(s): three records: (RC, 30 January 2016 [1]), in Martino and Tralongo (2021); (KR, 27 March 2021 and 30 March 2022 [2]), by Mario Pucci.

#### Oenanthe hispanica

A5FFA4A9-1DB3-52D3-896F-B220A6FD835B

##### Conservation status

DD

##### Distribution

Calabria, southern Italy

##### Notes

Code: A10.

#### Oenanthe pleschanka

55DDCBA2-AE49-5AD3-8AA9-80C25D6EEAE8

##### Distribution

Calabria, southern Italy

##### Notes

Code: A30; Annex I Birds Directive (2009/147/EC); Accidental record(s): two records: (CS 1988) in Scebba et al. (1993); (RC 1976) by Giuseppe Martino, a mounted specimen in a private collection.

#### Oenanthe leucura

3554DA0E-0A27-52CB-A69F-F31FB0B0E55B

##### Distribution

Calabria, southern Italy

##### Notes

Code: A30; Annex I Birds Directive (2009/147/EC); Accidental record(s): one record (CS 1999), in Scebba et al. (1993).

#### Regulus regulus

212D9FF9-2E7D-52B9-9BFD-EB8419B2FBC6

##### Conservation status

LC

##### Distribution

Calabria, southern Italy

##### Notes

Code: A11.

#### Regulus ignicapilla

F69E2851-A2CB-5508-B3FA-5C2017EF8724

##### Conservation status

LC

##### Distribution

Calabria, southern Italy

##### Notes

Code: A11.

#### Bombycilla garrulus

12EB01C5-587D-5206-96E5-CC4E8C2FD766

##### Distribution

Calabria, southern Italy

##### Notes

Code: A30; Accidental record(s): one record (RC 1970), in Scebba et al. (1993).

#### Prunella collaris

BDF5C646-8912-5F9E-8B42-8B465FEA903B

##### Conservation status

LC

##### Distribution

Calabria, southern Italy

##### Notes

Code: A10.

#### Prunella modularis

10088739-00E5-5C26-BA48-8A061077CFCB

##### Conservation status

NT

##### Distribution

Calabria, southern Italy

##### Notes

Code: A10.

#### Passer italiae

466497DB-239C-5290-B8A2-76A587709A38

##### Conservation status

VU

##### Distribution

Calabria, southern Italy

##### Notes

Code: A11.

#### Passer hispaniolensis

DEE1C3DC-D719-5786-8760-FB6D1D5F9745

##### Conservation status

VU

##### Distribution

Calabria, southern Italy

##### Notes

Code: A10.

#### Passer montanus

234B4C30-F4CE-522D-9854-A8818B9C1E90

##### Conservation status

NT

##### Distribution

Calabria, southern Italy

##### Notes

Code: A11.

#### Petronia petronia

739FF64F-5EC3-56F9-89C2-C9E532671D18

##### Conservation status

LC

##### Distribution

Calabria, southern Italy

##### Notes

Code: A11.

#### Anthus trivialis

9C17B4E2-11ED-57B1-8E05-70B0180947ED

##### Conservation status

LC

##### Distribution

Calabria, southern Italy

##### Notes

Code: A11.

#### Anthus cervinus

9FCFD363-4E40-5E76-8BD0-657BC2224763

##### Distribution

Calabria, southern Italy

##### Notes

Code: A10.

#### Anthus pratensis

1F8959A0-7BF2-5CBB-BA8F-0120C9AED285

##### Conservation status

NA

##### Distribution

Calabria, southern Italy

##### Notes

Code: A10.

#### Anthus spinoletta

0747A33E-13D5-54E0-83C1-90EAAA5B1442

##### Conservation status

LC

##### Distribution

Calabria, southern Italy

##### Notes

Code: A10.

#### Anthus richardi

FD519D79-345E-54C0-A84B-9F49F52F78E7

##### Distribution

Calabria, southern Italy

##### Notes

Code: B40.

#### Anthus campestris

055B0D95-5B50-51D6-B740-C6A7509A87E6

##### Conservation status

VU

##### Distribution

Calabria, southern Italy

##### Notes

Code: A11; Annex I Birds Directive (2009/147/EC).

#### Motacilla flava

3E273D77-9223-5500-8941-A75C0614B1E8

##### Conservation status

NT

##### Distribution

Calabria, southern Italy

##### Notes

Code: A11.

#### Motacilla flava thunbergi

C77DB241-E67F-5520-BFFB-90019AC97495

##### Distribution

Calabria, southern Italy

##### Notes

Code: A10.

#### Motacilla flava flava

F8909F7C-6701-5C3C-B313-EF60FC9F6935

##### Distribution

Calabria, southern Italy

##### Notes

Code: A10.

#### Motacilla flava feldegg

5B7CA5EA-14DA-59F1-A4B2-D7CF09FE6F44

##### Distribution

Calabria, southern Italy

##### Notes

Code: A10.

#### Motacilla flava cinereocapilla

8ED740B7-D193-539B-8DAF-C83ADD65C5C9

##### Distribution

Calabria, southern Italy

##### Notes

Code: A11.

#### Motacilla cinerea

1132067B-BEA9-5623-9585-59ED0E480955

##### Conservation status

LC

##### Distribution

Calabria, southern Italy

##### Notes

Code: A11.

#### Motacilla alba

F8B41189-8DF4-555E-891D-40F264537AAB

##### Conservation status

LC

##### Distribution

Calabria, southern Italy

##### Notes

Code: A11.

#### Fringilla coelebs

EB192B73-148F-5A5C-A2B7-583A3E6C7793

##### Conservation status

LC

##### Distribution

Calabria, southern Italy

##### Notes

Code: A11.

#### Fringilla montifringilla

A67A8A95-E4EC-5FEF-A056-B0CB77113C5C

##### Conservation status

NA

##### Distribution

Calabria, southern Italy

##### Notes

Code: A10.

#### Coccothraustes coccothraustes

50FA5866-D230-505A-B678-6438B32B4415

##### Conservation status

LC

##### Distribution

Calabria, southern Italy

##### Notes

Code: A10.

#### Pyrrhula pyrrhula

50034E1D-99C1-5E9D-A117-4D4B3DDAFEB8

##### Conservation status

LC

##### Distribution

Calabria, southern Italy

##### Notes

Code: A11.

#### Bucanetes githagineus

0B8FDB4B-D5FB-50CF-BAC3-4DCEED344FE8

##### Distribution

Calabria, southern Italy

##### Notes

Code: A30; Annex I Birds Directive (2009/147/EC); Accidental record(s): one record (RC, 24 March 2018), by Giuseppe Martino.

#### Chloris chloris

2DDBFCA3-9BC4-5F47-B7E3-3045A7854C1E

##### Conservation status

VU

##### Distribution

Calabria, southern Italy

##### Notes

Code: A11.

#### Linaria cannabina

D32593F6-B668-5AAA-A11C-F70E623EA3EB

##### Conservation status

NT

##### Distribution

Calabria, southern Italy

##### Notes

Code: A11.

#### Acanthis flammea

C59338E7-114A-5B34-879B-B95BC276EEA2

##### Conservation status

EN

##### Distribution

Calabria, southern Italy

##### Notes

Code: A30; Accidental record(s): one record (date and locality unknown), in Scebba et al. (1993).

#### Loxia curvirostra

8F68B8C5-6E52-5CC8-B6E0-2D853503703B

##### Conservation status

LC

##### Distribution

Calabria, southern Italy

##### Notes

Code: A11.

#### Carduelis carduelis

2572B5F1-668B-5AD1-AFA2-5670772A3B2B

##### Conservation status

NT

##### Distribution

Calabria, southern Italy

##### Notes

Code: A11.

#### Serinus serinus

864241C4-22A6-59BD-B932-528057D93493

##### Conservation status

LC

##### Distribution

Calabria, southern Italy

##### Notes

Code: A11.

#### Spinus spinus

2AF0F013-453F-504F-B8BB-E9A59E615B18

##### Conservation status

LC

##### Distribution

Calabria, southern Italy

##### Notes

Code: A11.

#### Emberiza melanocephala

2D7E8915-41D3-5C54-908B-AD28A3BF423D

##### Conservation status

DD

##### Distribution

Calabria, southern Italy

##### Notes

Code: A11.

#### Emberiza bruniceps

9ED4B2C0-FA38-5C4C-AA2C-F7C2A161E9A0

##### Distribution

Calabria, southern Italy

##### Notes

Code: A30; Accidental record(s): one record (RC 1979), by Giuseppe Martino, a mounted specimen in a private collection.

#### Emberiza calandra

49AB7767-2372-5336-B4B7-C90C02E0E548

##### Conservation status

LC

##### Distribution

Calabria, southern Italy

##### Notes

Code: A11.

#### Emberiza cia

14680745-09E2-53DB-8879-C1A350E30B3B

##### Conservation status

LC

##### Distribution

Calabria, southern Italy

##### Notes

Code: A11.

#### Emberiza hortulana

2915ED2D-B51A-59B4-A0A9-A9B2479B9601

##### Conservation status

DD

##### Distribution

Calabria, southern Italy

##### Notes

Code: A11; Annex I Birds Directive (2009/147/EC).

#### Emberiza caesia

BF85B170-A0DA-52D4-9337-2CD7666CB8A3

##### Distribution

Calabria, southern Italy

##### Notes

Code: B40; Annex I Birds Directive (2009/147/EC).

#### Emberiza cirlus

0708D2C9-E010-5860-8F32-88C227F99328

##### Conservation status

LC

##### Distribution

Calabria, southern Italy

##### Notes

Code: A11.

#### Emberiza citrinella

0B62E4E0-CB7A-5ED5-9580-D7C884B6093B

##### Conservation status

VU

##### Distribution

Calabria, southern Italy

##### Notes

Code: A11.

#### Emberiza leucocephalos

DC159CA2-A5D4-580E-81EA-C3CB095BD689

##### Distribution

Calabria, southern Italy

##### Notes

Code: A30; Accidental record(s): one record (RC 1975), by Giuseppe Martino, a mounted specimen in a private collection.

#### Emberiza schoeniclus

82EF90A0-DFD2-5FE2-8AFA-B0FE0A63065A

##### Conservation status

CR

##### Distribution

Calabria, southern Italy

##### Notes

Code: A10.

## Analysis

### Results

The updated checklist contains 363 species and nine subspecies of birds, with records confirmed up to 31 December 2025; subspecies are listed below their respective parent species and are not assigned a progressive number. Of the 363 species included, 336 are assigned to AERC category A (apparently wild origin, recorded at least once since 1950), 12 to category B (only historical records, pre-1950), seven to category C (introduced or escaped, with self-sustaining populations), five to category AC (intermediate between A and C) and three to category E (introduced or escaped, not meeting category C criteria).

General status codes refer to 258 regular taxa, 20 irregular, 64 accidental/vagrant and 21 historical. Breeding status was assigned to 143 taxa confirmed as regular breeders during the last decade, eight irregular breeders, three occasional breeders and four former breeders no longer reproducing in the region. For the remaining 205 species, no confirmed breeding evidence is available for Calabria.

A total of 125 species are listed in Annex I of the EU Birds Directive (Directive 2009/147/EC), highlighting the conservation relevance of the regional avifauna. The national IUCN category from the Red List of breeding birds in Italy (Gustin et al. 2021) was retrieved for 230 species: nine are Critically Endangered (CR), 21 Endangered (EN), 32 Vulnerable (VU), 28 Near Threatened (NT), 124 Least Concern (LC), 12 Data Deficient (DD) and four Regionally Extinct (RE).

### Comparison with the previous regional checklist

Compared with the previous regional checklist of 320 species (Scebba et al. 1993), the present compilation includes 43 additional species. After harmonising the nomenclature of the two lists through a verified mapping of synonyms (resolving 28 genus- or species-level name changes published since 1993), none of the species reported by Scebba et al. (1993) was excluded from the present list. Consequently, these 43 additions represent a true net gain in regional species richness.

The new entries are distributed across 12 orders, with Passeriformes accounting for the largest share (15 species, 35% of the total), followed by Charadriiformes (8), Anseriformes (7), Accipitriformes (3), Pelecaniformes (2), Galliformes (2) and one species each in Bucerotiformes, Caprimulgiformes, Columbiformes, Gruiformes, Podicipediformes and Psittaciformes. At the family level, the largest contributions come from Anatidae (7 species), Scolopacidae (4) and Phylloscopidae (4).

Of the 43 new species, 34 belong to AERC category A (wild origin), six to category C (introduced or escaped, naturalised) and three to category E (escapes). The general status of these additions shows a strong skew towards accidental occurrences: 35 species (81%) are classified as accidental/vagrant, five as irregular and only three as regular (*Larus michahellis*, *Stercorarius pomarinus* and *Passer hispaniolensis*). Breeding has been confirmed for four of the new species: *Larus michahellis* (regular breeder), *Apus caffer* (irregular), *Porphyrio porphyrio* (occasional) and the naturalised *Psittacula krameri* (irregular).

The accidental additions reflect the geographic position of Calabria along the central Mediterranean flyway, with vagrants originating from a wide range of biogeographic regions: ten species are of Siberian or Asian origin (mostly Phylloscopidae and Muscicapidae), nine are of African or Middle-Eastern origin, five from northern Europe or the Arctic and one (*Calidris melanotos*) of Nearctic origin. The remaining ten additions are of broadly Eurasian or Mediterranean provenance, including several naturalised or introduced taxa.

## Discussion

Here, we provide an updated checklist of the birds of Calabria that synthesises available information in a standardised and verifiable format, fully compatible with the taxonomy and coding scheme of the national CISO–COI checklist (Baccetti et al. 2021). Compared with the previous regional checklist (Scebba et al. 1993), the present compilation reflects more than thirty years of accumulated fieldwork, citizen-science contributions and improvements in observer coverage across the region.

### Drivers of the increase since 1993

The 43 species added since the historical checklist do not reflect a uniform process. The strong skew towards accidental occurrences (35 of 43, 81%) indicates that most additions result from improved observer effort and more systematic documentation of vagrancy rather than from genuine range expansions. In particular, several Siberian vagrants (e.g. *Phylloscopus proregulus*, *P. fuscatus, P. inornatus*, *Tarsiger cyanurus*, *Turdus obscurus*) have been recorded within the Phylloscopidae and Muscicapidae groups. These species would have been very difficult to detect without targeted ringing activity at coastal stations, such as Punta Alice (KR) and San Michele di Cetraro (CS).

A second component consists of Anatidae (seven new species) and Scolopacidae (four new species), reflecting better coverage of coastal wetlands and improved identification skills amongst regional observers. Six species belong to AERC category C and reflect naturalisation processes occurring at a national or international scale rather than at a purely regional one (e.g. *Threskiornis aethiopicus, Psittacula krameri, Alectoris chukar*).

Only a small number of additions involve confirmed breeding in the region, including the regional colonisation by *Larus michahellis* (regular breeder), the occasional breeding of *Porphyrio porphyrio*, the recent naturalisation of *Psittacula krameri* and the irregular nesting of *Apus caffer*, the latter representing the first documented breeding of the species in Italy and the entire central Mediterranean Basin (Pucci et al. 2022).

### Biogeographic significance

Calabria, the southernmost mainland Italian region, occupies a strategic position between the Tyrrhenian and Ionian basins and faces Sicily and the African coast. Therefore, it plays an important role in bird migration and vagrancy along the central Mediterranean flyway, as demonstrated by the heterogeneous biogeographic origin of vagrants reported from the region: approximately one quarter of the newly-reported taxa were of Siberian or central Asian origin, a quarter were of African or Middle Eastern origin and a smaller, but still significant fraction was of Arctic, northern European or Nearctic origin (Massa et al. 2021). This diversity of provenance, which holds true also for other southern Italian regions and Sicily (Massa et al. 2021), confirms Calabria as a geographical area where different migration systems converge. The evidence of breeding by *Apus caffer* at an inland site in the province of Crotone, here confirmed, is the first documentation of nesting by this species in Italy and the central Mediterranean Basin at large (Pucci et al. 2022) and epitomises Calabria as a putative bridgehead for Afro-Iberian taxa colonising Europe.

### Conservation relevance

The high number of Annex I species (125 of 363, 34%) and the substantial representation of species included in threatened categories of the national IUCN Red List (9 CR, 21 EN, 32 VU) underline the regional conservation importance of Calabrian avifauna. The Critically Endangered species recorded in Calabria are particularly significant because most of them are wetland-dependent taxa (*Porzana porzana*, *Zapornia parva*, *Chlidonias niger*, *Acrocephalus schoenobaenus*, *Emberiza schoeniclus*), alongside emblematic large raptors (*Pandion haliaetus*, *Gypaetus barbatus*, *Neophron percnopterus*) and a passerine of open structured habitats (*Sylvia nisoria*), reflecting the vulnerability of both freshwater and coastal habitats and of the regional populations of cliff-nesting and scavenging raptors. Wetland-dependent species more broadly (Anatidae, Ardeidae, Charadriiformes) account for a relevant share of the regional avifauna and depend on a limited and fragmented network of coastal and inland wetlands. The Calabrian network of Natura 2000 sites partially overlaps with key sites for these taxa, but conservation effectiveness varies considerably across sites and requires sustained monitoring.

### Comparison with neighbouring regions

The 363 species recorded in Calabria can be compared with other regional checklists of southern Italy and adjacent areas. Sicily, with a much longer ornithological tradition, has recorded 437 species since the mid-19^th^ century (Massa et al. 2021), reflecting both its larger surface area and the much greater observer effort historically devoted to the island. In Pantelleria Island alone (Sicilian Channel), 261 bird species have been spotted (Corso et al. 2012), illustrating how even small Mediterranean territories can accumulate substantial vagrant records under sustained observer effort.

The Italian national checklist comprises 551 species and 702 taxa (Baccetti et al. 2021), of which Calabria holds approximately two-thirds of the national diversity. This high proportion is in line with what would be expected given the region's geographic extent and habitat heterogeneity.

### Remaining gaps and future priorities

Despite the substantial improvement over the 1993 checklist, several knowledge gaps remain. Inland mountain areas (Pollino, Sila, Aspromonte) are still under-surveyed compared with coastal sites and standardised data on breeding densities and population trends are lacking for most species. Pelagic seabirds, nocturnal migrants and high-altitude breeders deserve dedicated monitoring programmes. The development of a regional breeding bird atlas integrating standardised square-based surveys would constitute the natural next step. Coordinated wintering counts at coastal wetlands, expansion of the network of constant-effort ringing stations and integration with national citizen-science platforms (ORNITHO.it, eBird, iNaturalist) under quality-controlled validation procedures will be essential to consolidate the regional knowledge base. The long list of accidental and vagrant taxa documented here also underlines the need for continued coordination amongst observers under shared methodological standards, of the kind now ensured by the Stazione Ornitologica Calabrese.

## Figures and Tables

**Table 1.** Definitions of AERC categories and the occurrence/breeding status codes used in the checklist, following Baccetti et al. (2021).

**Class**	**Code**	**Definition**
**AERC category**	A	Apparently wild origin; recorded at least once since 1950.
**AERC category**	B	Apparently wild origin; recorded at least once between 1800 and 1949.
**AERC category**	C	Introduced or escaped; has formed at least one self-sustaining breeding population; also includes natural colonisation from such populations outside Italy.
**AERC category**	D	Wild origin possible, but uncertain; may be due to escape/release, passive transport etc., or cannot be placed in other categories.
**AERC category**	E	Introduced or escaped; does not meet the criteria for category C.
**General status**	1	Regular: recorded in at least 9 of the last 10 years.
**General status**	2	Irregular: recorded > 10 times and in ≥ 6 years since 1950, but in < 9 of the last 10 years.
**General status**	3	Accidental: recorded 1–10 times or in 1–5 years since 1950.
**General status**	4	Historical: recorded at least once, but not since 1950.
**Breeding status**	1	Regular: breeding confirmed in at least 9 of the last 10 years.
**Breeding status**	2	Irregular: breeding confirmed in 4–8 of the last 10 years.
**Breeding status**	3	Occasional: breeding confirmed only in 1–3 of the last 10 years (or more).
**Breeding status**	4	Historical: formerly breeding (apparently regular at some period), but not in the last 10 years.
**Breeding status**	0	No confirmed breeding evidence in Calabria.
